# Integrative analysis of a novel super-enhancer-associated lncRNA prognostic signature and identifying LINC00945 in aggravating glioma progression

**DOI:** 10.1186/s40246-023-00480-w

**Published:** 2023-03-31

**Authors:** Zhihao Yang, Yinfei Zheng, Haoyuan Wu, Han Xie, Jiajia Zhao, Zhigang Chen, Lianxin Li, Xiaoyu Yue, Bing Zhao, Erbao Bian

**Affiliations:** 1 Department of Neurosurgery, The Second Affiliated Hospital of Anhui Medical University, 678 Fu Rong Road, Hefei, 230601 Anhui Province China; 2 Cerebral Vascular Disease Research Center, Anhui Medical University, 678 Fu Rong Road, Hefei, 230601 Anhui Province China

**Keywords:** Super-enhancer, lncRNA, Prognostic signature, Immunotherapy, Glioma

## Abstract

**Background:**

Super-enhancers (SEs), driving high-level expression of genes with tumor-promoting functions, have been investigated recently. However, the roles of super-enhancer-associated lncRNAs (SE-lncRNAs) in tumors remain undetermined, especially in gliomas. We here established a SE-lncRNAs expression-based prognostic signature to choose the effective treatment of glioma and identify a novel therapeutic target.

**Methods:**

Combined analysis of RNA sequencing (RNA-seq) data and ChIP sequencing (ChIP-seq) data of glioma patient-derived glioma stem cells (GSCs) screened SE-lncRNAs. Chinese Glioma Genome Atlas (CGGA) and The Cancer Genome Atlas (TCGA) datasets served to construct and validate SE-lncRNA prognostic signature. The immune profiles and potential immuno- and chemotherapies response prediction value of the signature were also explored. Moreover, we verified the epigenetic activation mechanism of LINC00945 via the ChIP assay, and its effect on glioma was determined by performing the functional assay and a mouse xenograft model.

**Results:**

6 SE-lncRNAs were obtained and identified three subgroups of glioma patients with different prognostic and clinical features. A risk signature was further constructed and demonstrated to be an independent prognostic factor. The high-risk group exhibited an immunosuppressive microenvironment and was higher enrichment of M2 macrophage, regulatory T cells (Tregs), and Cancer-associated fibroblasts (CAFs). Patients in the high-risk group were better candidates for immunotherapy and chemotherapeutics. The SE of LINC00945 was further verified via ChIP assay. Mechanistically, BRD4 may mediate epigenetic activation of LINC00945. Additionally, overexpression of LINC00945 promoted glioma cell proliferation, EMT, migration, and invasion in vitro and xenograft tumor formation in vivo.

**Conclusion:**

Our study constructed the first prognostic SE-lncRNA signature with the ability to optimize the choice of patients receiving immuno- and chemotherapies and provided a potential therapeutic target for glioma.

**Supplementary Information:**

The online version contains supplementary material available at 10.1186/s40246-023-00480-w.

## Introduction

Gliomas are the most common malignant primary brain tumor, accounting for about 44% of central nervous system (CNS) tumors [1]. The World Health Organization (WHO) classifies gliomas into four categories, namely grade I, II, III, and IV [2]. Glioblastoma (GBM) is the highest glioma grade and the most aggressive subtype. The median overall survival (OS) of GBM patients is approximately 15 months despite the comprehensive treatment with surgery followed by radiation and chemotherapy [3]. Emerging bionomics studies have improved diagnostic and therapeutic strategies for GBM, but satisfactory results have yet to be achieved due to its complex pathogenesis and molecular heterogeneity. Hence, it is desirable to explore novel molecular markers to accurately assess glioma prognosis and develop more efficient treatment strategies.

The tumor microenvironment (TME), comprising various immune cells, stromal cells, fibroblasts, endothelial cells, blood vessels, and secreted factors, profoundly impacts tumor initiation and development, leading to poor prognosis [4]. In recent years, immunotherapy, characterized by activating tumor-infiltrating lymphocytes (TILs), killing tumor cells, and inhibiting tumor metastasis and recurrence, is the most likely treatment option for long-term survival and has become a hot topic in the therapy of many cancers [5, 6]. Accumulating evidence indicates that the degree of response to immunotherapy depends partly on the abundance of tumor-infiltrating immune cells (TIICs) [7]. Among immunotherapy, immune checkpoint blockade (ICB) was the most widely studied [8]. Compared with conventional therapies, the application of immune checkpoint inhibitors (ICIs) represented by anti-PD1, anti-PD-L1, and anti-CTLA4 in several cancers has presented better therapeutic effects [9, 10]. ICB is expected to be a promising immunotherapy strategy for glioma.

Abnormal gene expression caused by transcriptome reprogramming, acting as the crucial characteristic of cancer, contributes to tumor initiation, progression, and metastasis [11]. Super-enhancers (SEs), clustered or stretched enhancers, harbor high-density transcription factors and transcriptional coactivators to provide synergistic gene activation [12]. Compared to typical enhancers, SEs elicit more potent effects. They are enriched in crucial genes which define cell identity and differentiation and promote the expression of these genes [13]. Recent studies have demonstrated that cancer cells acquire cancerous phenotype dependent on the aberrant transcription of oncogenes driven by SEs [14]. Importantly, SEs constantly propelled high-level expression of tumor-promoting long non-coding RNAs (lncRNAs) [15]. Thus, it is of great interest to characterize SEs that drive oncogenic lncRNA in the pathogenesis of glioma.

LncRNAs are generally defined as transcripts that are more than 200 nucleotides in length and cannot encode proteins. According to reports, lncRNA participates in extensive vital biological functions, and dysregulated expression of lncRNAs leads to disease phenotypes, including malignant tumors [16]. Mechanistically, most studies focus on the downstream regulatory functions of lncRNAs, including interaction with proteins/RNAs, epigenetic regulation, transcriptional regulation, and other regulatory processes, whereas few investigations are directed toward assessing how lncRNAs are regulated [17]. At the transcription level, SEs mediate the dysregulation of lncRNAs in several types of cancer. Super-enhancer-associated lncRNAs (SE-lncRNAs) participate in many cancer-related biological processes, such as angiogenesis, proliferation, invasion, and metastasis. Peng et al. reported that SE-lncRNA HCCL5 activated by ZEB1 promotes the malignant progression of hepatocellular carcinoma [18]. In colorectal cancer, SE-lncRNA AC005592.2 promotes tumor progression by regulating OLFM4 [19]. However, research on SE-lncRNAs and glioma is still rare. In recent years, with the progress of bioinformatics and genome sequencing technology, various types of lncRNAs-related models have been widely reported to evaluate the prognosis of tumors, including m6A-related lncRNAs, immune-related lncRNAs [20, 21]. However, the clinical and biological significance of the SE-lncRNA signature has yet to be explored.

In this research, we first identified 6 SE-lncRNAs by combining RNA-seq and ChIP-seq data analysis. According to the expression profile of the 6 SE-lncRNAs in the Chinese Glioma Genome Atlas (CGGA) and The Cancer Genome Atlas (TCGA) datasets, we identified three glioma subgroups with disparate clinicopathological and prognostic features and developed a risk signature serving as an independent indicator for prognosis of gliomas. Additionally, immunophenoscore (IPS), tumor purity, immune cell infiltration, immunotherapy, and chemotherapy associated with our signature in glioma were entirely investigated. Subsequent experiments revealed the epigenetic activation mechanism of SE-LINC00945. The overexpression of LINC00945 facilitated glioma cell proliferation, epithelial-mesenchymal transition (EMT), migration, invasion, and tumor growth in xenograft tumor models in nude mice. In general, these findings reveal that the SE-lncRNA signature will better evaluate the prognosis of glioma and choose effective treatment, and LINC00945 may act as a novel target for glioma treatment.

## Materials and methods

### Tissue specimen

After obtaining the patient’s informed consent, tissue samples were collected at the Department of Neurosurgery, the Second Affiliated Hospital of Anhui Medical University (Hefei, China). Glioma patients undergoing surgical resection were further pathologically confirmed. A total of 8 normal brain tissues and 12 glioma tissues were stored at −80 ℃. Additional file 6: Table S1 lists the patient’s clinical information and primary tumor characteristics. The Research Ethics Committee of the Second Affiliated Hospital of Anhui Medical University approved this study.

### Patients and datasets

The RNA-seq data and ChIP-seq data (GSE119776) of 43 glioma patient-derived GSCs and 9 neural stem cells (NSCs) were retrieved from the Gene Expression Omnibus (GEO) database. The RNA-seq data (n = 620) and corresponding clinical information (n = 619) in the CGGA database were used as the training set. Likewise, the validation set derived from the TCGA database, including RNA-seq data of 670 glioma patients and clinical data (n = 665), was downloaded.

### Consensus clustering analysis

The “ConsensusClusterPlus” R package was utilized to perform cluster classification to divide glioma patients into different subgroups. The optimum amount of clusters was assessed by implementing the cumulative distribution function (CDF) and consensus matrices [22].

### Establishment of prognostic signature

The LASSO Cox regression algorithm [23] was conducted to construct a prediction model. The minimum criteria identified 6 SE-lncRNAs and their coefficients, selecting the optimal penalty parameter (λ) related to the minimum tenfold cross-validation in the training set. Then, the corresponding risk score of each patient was obtained, and the formula is as follows:

$$Riskscore = \mathop \sum \limits_{x = 1}^{n} coef\left( x \right) \times exp\left( x \right).$$ 

In this equation, coef(x) and exp(x) were the regression coefficients and expression values of lncRNAs, respectively. Finally, glioma patients were divided into high- and low-risk groups based on the median risk score in the training or validation sets.

### Functional analysis

Gene Ontology (GO) and Kyoto Encyclopedia of Genes and Genomes (KEGG) analyses were used to perform functional enrichment analysis of our risk signature utilizing the R package “clusterProfiler”. Gene Set Enrichment Analysis (GSEA) was implemented to reveal the functions associated with the 6 SE-lncRNAs via GSEA software.

### Immune microenvironment characterization

The R package “ESTIMATE” was used to assess tumor purity by estimating markers belonging to stromal and immune cells [24]. The immunophenoscore (IPS) quantifies four immunophenotypes using abundant immune response/toleration markers, including antigen presentation, effector cells, checkpoints, and suppressor cells. Moreover, the z-score, the summary of four categories, represents the immunogenicity of the corresponding sample [25]. At the same time, the CIBERSORT [26], single-sample gene set enrichment analysis (ssGSEA) [27], QUANTISEQ [28], MCPcounter [29], and EPIC [30] algorithms performed to evaluate immune cell infiltration in two risk groups, were presented with a heatmap.

### Prediction for response to immunotherapy and chemotherapy

Subclass mapping was employed to assess the clinical response to ICI between two risk groups [31]. The gene expression matrices of glioma patients in two subgroups were compared to the expression profile recording the expression data of 47 melanoma patients treated with ICI against PD1 or CTLA4. Genomics of Drug Sensitivity in Cancer (GDSC) was utilized to predict the chemotherapeutic response for every patient [32]. The R package “pRRophetic” was utilized to assess the samples’ half-maximal inhibitory concentration (IC50) by ridge regression. The tenfold cross-validation based on the GDSC training set was performed to examine the prediction accuracy.

### Cell culture and treatment

Normal astrocyte HEB and glioma cell lines LN18, T98G, U251, SF126, and SNB19 were purchased from the Chinese Academy of Sciences. PN12 was the patient-derived glioblastoma cell obtained from surgical specimens, which has been reported in our previous research [33]. Cells were cultured using DMEM (Gibco, USA), including 10% FBS (Gibco, USA) in an incubator at 37 °C and 5% CO_2_. The culture medium was changed every two days, and cell passaging was conducted once the density reached 80%. We purchased JQ1 from MedChemExpress (Princeton, NJ). For knockdown experiments, human-specific small interfering RNA (siRNA) against MED1 and negative control (Genepharma Technology, Shanghai, China) were transfected into U251 and T98G glioma cells utilizing the transfection reagent jetPRIME (Poly plus-transfection®). The siRNA target sequences for MED1 were as follows: si-MED1-1 (5’-GGAGGAAAGCUGAAACCAUTT-3’, 5’-AUGGUUUCAGCUUUCCUCCTT-3’), and si-MED1-2 (5’-GCUCAUGAACCUUCUUAAATT-3’, 5’-UUUAAGAAGGUUCAUGAGCTT-3’). Stable cell lines were established after being screened with puromycin.

### ChIP-seq analysis

Aspera was used to download the raw fastq file from the GEO database (GSE119776). Reads were mapped to the reference utilizing Bowtie2, and samtools were employed to sort and index reads. BigWig files were derived from bam files of every sample utilizing genomeCoverageBed and bedGraphToBigWig. In addition, peak calling was performed utilizing macs14. The Rank Ordering of Super-Enhancers (ROSE) algorithm was applied to identify SEs (Stitching distance: 12.5 kb). DeepTools were implemented for downstream analysis, and IGV genome browser was used to perform visual inspection.

### Chromatin immunoprecipitation (ChIP)

ChIP assay was performed utilizing SimpleChIP Plus Sonication Chromatin IP Kit (Cell Signaling Technology) following the operation instruction of the manufacturer. In brief, the chromatin of glioma cells was incubated with 2 μg anti-H3K27ac (Abcam, ab4729). Additional file 6: Table S2 shows the specific primers used to detect immunoprecipitated DNA through RT-qPCR.

### RNA extraction and real-time quantitative PCR

Total RNA of tissues and cells was obtained with TRIzol reagent (Invitrogen; Thermo Fisher Scientific, Inc.), and the concentration, as well as the quality of RNA, were detected by the nanodrop spectrophotometer (IMPLEN GmbH) in 260/280 nm absorbance. Then, RT-qPCR was conducted utilizing SYBR Green mix (TaKaRa Biotechnology, China) with primers in the ABI 7500 Real-Time PCR System (Applied Biosystems) after reverse transcription of RNA to cDNA. Finally, the 2-DeltaDeltaCt method was used to calculate relative quantification, and GAPDH served as the reference. Additional file 6: Table S2 presents the primers used in the study.

### Western blotting

The total protein of treated glioma cells was obtained using RIPA lysis buffer (Beyotime, China) and then separated by SDS-PAGE. The protein was transferred onto the PVDF membrane (MilliporeCorp, USA) and incubated with primary antibodies overnight after blocking for 3 h in the blocking solution (5% skim milk in TBST). Subsequently, TBST was used to wash membranes three times, adding the secondary antibody at the dilution of 1: 10,000 in TBST to incubate for 1 h. Finally, the ECL chemiluminescence kit (Thermo Scientific) was utilized to visualize proteins after washing three times. The antibodies used are as follows: anti-β-actin (Abcam, ab8226), anti-N-cadherin (CST, #13,116), anti-ZEB1 (CST, #3396), and anti-ZEB2 (BOSTER, BA2872-2), anti-BRD4 (Abcam, ab128874), and anti-MED1 (BETHYL, A300-793A-T).

### Cell counting kit (CCK)-8 assay

After treatment, LN18 and PN12 cells were inoculated into 96-well plates in quantities of 1000 cells per well for 0 h, 24 h, 48 h, and 72 h. Subsequently, 10 μl working solution (Dojindo, Japan) was added to the corresponding well. We conducted five repeats in different wells. After incubating for 2 h, the optical density (OD) value was read utilizing the microplate reader at 450 nm.

### Colony forming assay

The transfected LN18 and PN12 cells were seeded in 6-well plates with 600 cells per well, and single-cell colonies were formed after two weeks in an incubator. Then, cells were fixed with 4% paraformaldehyde for half an hour and 0.5% crystal violet stained colonies for another 30 min. More than 50 cells make up a clone. Finally, we counted the number of colonies utilizing the microscope (Olympus, Japan) and photographed them via a camera.

### Cell migration and invasion assays

2 × 10^4^ cells, suspended in serum-free medium, were counted and added into a Transwell chamber which was placed in a 24-well plate with 600ul DMEM containing 30% FBS and then cultured for 24 h for migration experiment. In the invasion experiment, we added 8 × 10^4^ cells to the chambers coated with matrix (Corning, 356234) and cultured them under the same conditions for 48 h. When the time was up, cells were treated with 4% paraformaldehyde for 30 min and then stained with 0.5% crystal violet for 15 min. Finally, after washing with water and wiping off the cells on the upper surface of the chambers, the cells were photographed with a microscope (Olympus, Japan).

### Tumor xenograft formation assay in vivo

We purchased 4-week-old BALB/c-nude mice from Jiangsu Jicukang Biotechnology Co., Ltd. (China), and a total of 14 mice were randomly divided into two groups (n = 7 per group). Experiments were performed strictly based on a protocol approved by the Animal Research Committee of Anhui Medical University. Mice were kept and handled following guidelines and recommendations. We suspended glioma cells in PBS/Matrigel at a density of 5 × 10^6^ cells/ml, and 100 μl cell suspension was subcutaneously injected into the flanks of nude mice. Tumor volumes were measured every 7 days and determined using the formula: V = 0.5 × L × W^2^ (L: length; W: width). 28 days after inoculation, tumor tissues were harvested and weighed for further study.

### Statistical analyses

All experiments were repeated at least three times, and the experimental data were expressed as the mean ± SD. Unpaired t-test or one‐way ANOVA was performed to compare continuous variables between groups. We conducted the Kaplan–Meier method to investigate the OS of each group and the chi-square test to analyze differences in clinicopathological characteristics. Pearson correlation coefficients were utilized to evaluate the relationship between variables, and we performed statistical analyses using GraphPad Prism or R software. *p < 0.05 indicates statistical significance.

## Results

### Identification of SE-LncRNAs

**Fig. 1:**
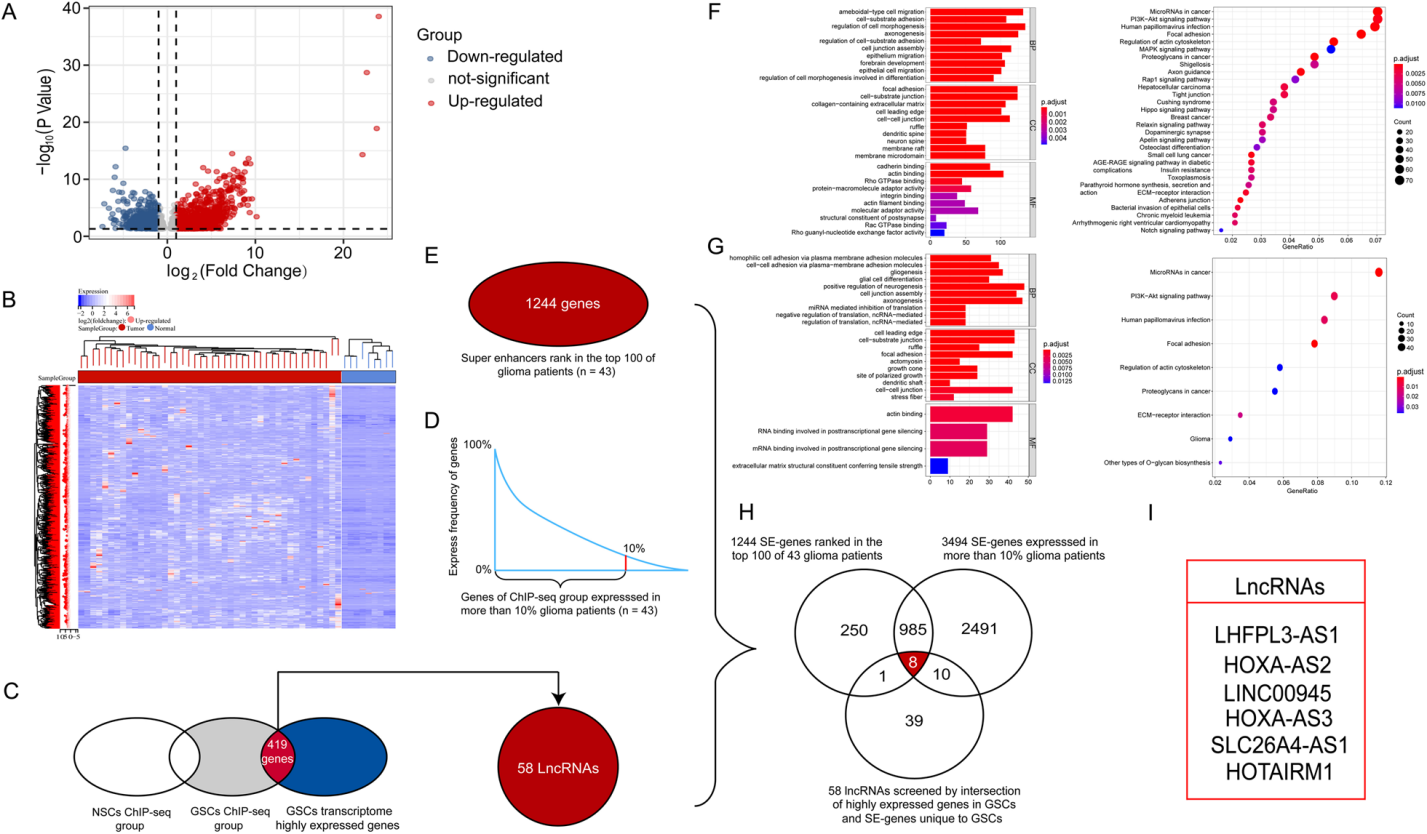
Identification of 6 SE-lncRNAs. **A** A volcano plot of 2441 differentially expressed SE-genes. **B** The heatmap shows expression levels of 1688 SE-genes which are highly expressed in GSCs. **C** Identification of 58 SE-lncRNAs. **D** The 3494 specific SE-genes were expressed in more than 10% of the 43 glioma patients. **E** Select each patient's top 100 genes in ChIP-seq, resulting in 1244 SE-genes. **F** GO and KEGG analyses of the 3494 SE-genes screened by frequency. **G** GO and KEGG analyses of the 1244 SE-genes screened by rank order. **H** 8 SE-lncRNAs were obtained by the intersection of 58 SE-lncRNAs with SE-genes obtained by frequency and ranking. **I** 6 SE-lncRNAs were finally screened by combining the RNA-seq data of TCGA and CGGA databases

As shown in the volcano plot, we first identified 2441 genes that were differentially expressed in GSCs and NSCs using |log2 FC|> 1 and *P* < 0.05 as cut-offs via RNA-seq data (Fig. 1A), and 1688 genes highly expressed in GSCs were displayed in a heatmap (Fig. 1B). Then, based on the H3K27ac ChIP-seq data of 43 glioma patients-derived GSCs, SEs were identified by the ROSE algorithm, and genes regulated by SEs were selected; subsequently, we obtained 9606 super-enhancer-associated genes (SE-genes) by merging these genes and removing duplicates. In the same way, 6054 SE-genes were yielded from the ChIP-seq data of NSCs. We analyzed these two sets of SE-genes by a Venn diagram, and 4553 SE-genes unique to GSCs were selected. The intersection of these 4553 SE-genes with 1688 genes screened by differential analysis yielded 419 SE-genes, and 58 SE-lncRNAs were further identified via gene annotation (Fig. 1C). Subsequently, we screened for genes based on the frequency and rank ordering of SE-genes in the ChIP-seq data of GSCs. The frequency condition was that SE-genes were present in more than 10% of all 43 glioma patients. We integrated SE-genes of GSCs and calculated the frequency of each gene in an EXCEL table. Genes with a frequency greater than or equal to 5 were selected, and 3494 SE-genes were obtained (Fig. 1D). As for rank order, we chose the top 100 SE-genes in ChIP-seq data of each patient's GSCs and got 1244 SE-genes after removing duplicate genes (Fig. 1E). We further performed GO and KEGG analyses on these two sets of SE-genes. Results indicated that both were associated with biological processes such as focal adhesion and cell–cell junction (Fig. 1F, G). Finally, the intersection of 58 SE-lncRNAs and SE-genes obtained by frequency and rank ordering obtained 8 SE-lncRNAs, and 6 SE-lncRNAs were eventually screened by combining with RNA-seq data in CGGA and TCGA databases (Fig. 1H, I).

### Consensus clustering of 6 selected SE-LncRNAs identified 3 clusters of glioma patients

**Fig. 2:**
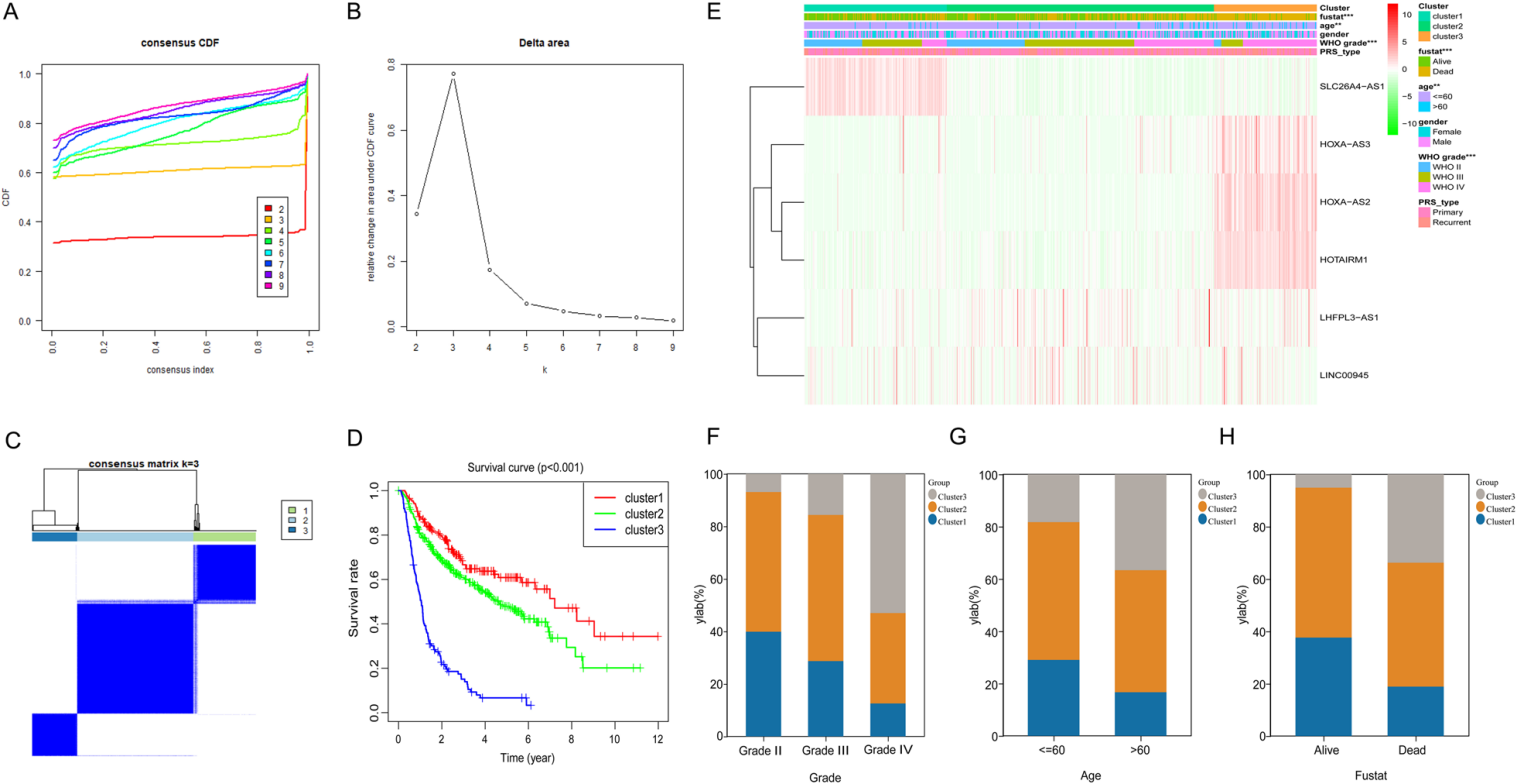
6 SE-lncRNAs classified glioma patients into clusters with discrepant OS and clinical features in the CGGA dataset. **A** CDF of consensus clustering from k = 2–9. **B** The area under the CDF curve was changed from k = 2 to 9. **C** The consensus clustering matrix revealed that glioma patients in CGGA dataset were divided into 3 clusters. **D** Survival differences among cluster 1/2/3. **E** Discrepant clinicopathologic features and 6 SE-lncRNA expression levels among cluster 1/2/3. **F**–**H** The clinicopathological features in 3 subgroups, including grade (**F**), age (**G**), and survival status (**H**). **p < 0.01, ***p < 0.001

Based on the expression similarity in 6 selected lncRNAs, k = 3 was an appropriate choice with clustering stability in the CGGA dataset (Fig. 2A–C). Additionally, patients in cluster 3 showed the shortest OS among the three subgroups (Fig. 2D). Then, we made a comparison of the clinicopathological features of 3 subgroups. Results revealed that lower grade, younger age, and alive status were mainly presented in the cluster 1 and 2 subgroups, while the cluster 3 subgroup was prominently related to GBM phenotype, older age at diagnosis, and dead status (Fig. 2E–H). At the same time, glioma patients of the same grade were distributed in different subgroups, indicating the heterogeneity of glioma of the same grade.

Similar results were observed in the TCGA database. Additional file 1: Fig. S1A-C suggested that the most appropriate choice was to divide the cases into 3 clusters. Moreover, the cluster 3 subgroup showed the shortest OS (Additional file 1: Fig. S1D). Combined with clinical information, the cluster 1 and 2 subgroups significantly correlated to lower grade glioma, alive status, and younger age. On the contrary, the cluster 3 subgroup consisted mainly of patients with GBM phenotypes, dead status, and older age (Additional file 1: Fig. S1E–H).

### The clinical information and prognostic impact of the risk signature constructed by 6 selected SE-LncRNAs in glioma

**Fig. 3:**
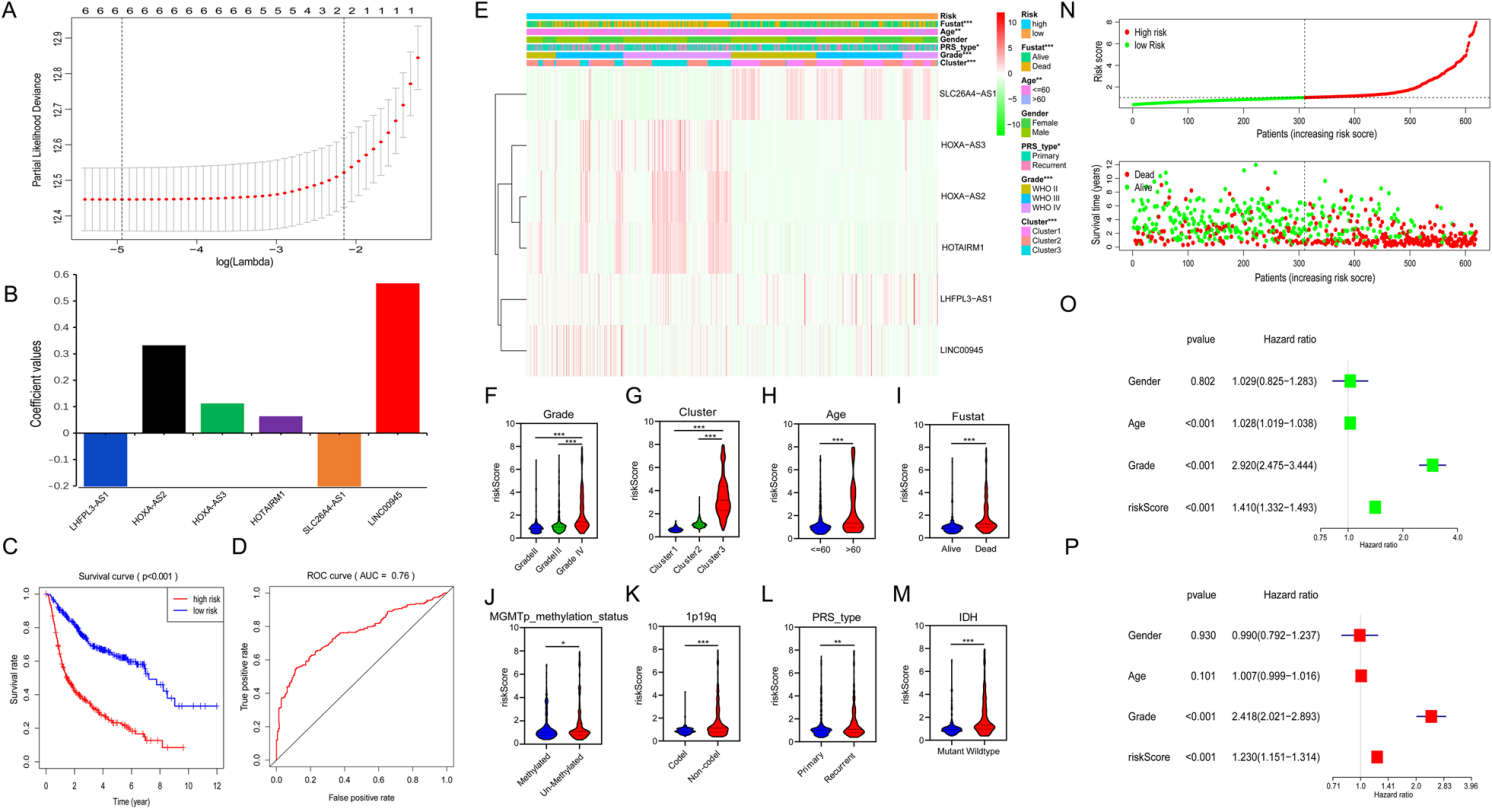
Construction of a 6 SE-lncRNAs risk model. **A** Cross-validation for adjusting parameter choice in LASSO regression analysis. **B** 6 SE-lncRNAs and the corresponding coefficients were shown. **C** Kaplan–Meier survival curves of two risk groups in the CGGA dataset. **D** The ROC curve indicates the sensitivity and specificity of the risk signature to predict prognosis in the training set. **E** The heatmap displayed the discrepant distribution of clinical features and expression of 6 SE-lncRNAs in low- and high-risk glioma patients. **F**–**M** The WHO grade (F), cluster 1/2/3 subgroups (**G**), age (**H**), fustat (**I**), MGMT promoter methylation status (**J**), 1p/19q codel status (**K**), PRS type (**L**), and IDH status (**M**) stratify the training set, and the distribution of risk scores was shown. **N** Surveying the risk model in CGGA dataset: distribution and survival status of glioma patients in low- and high-risk groups. **O**, **P** Univariate (**O**) and multivariate (**P**) analyses for the training set consisting of gender, age, grade, and risk score. *p < 0.05, **p < 0.01, ***p < 0.001

Next, the prognostic effect of 6 selected SE-lncRNAs was explored in gliomas. Firstly, LASSO Cox regression analysis of these lncRNAs was conducted to develop a risk signature in the CGGA training dataset (Fig. 3A). Figure 3B showed the corresponding coefficients, and then the risk score for each patient was calculated in both CGGA and TCGA datasets. With the median risk score as the threshold, two datasets classified glioma patients into low- and high-risk groups. We then compared the survival differences between the two groups, with patients in the low-risk group showing longer OS in both the training (Fig. 3C) and testing (Additional file 2: Fig. S2A) datasets. Furthermore, the AUC of ROC curves was 76% and 75.3% in training (Fig. 3D) and validation (Additional file 2: Fig. S2B) sets, respectively, which means that our model is accurate. These results manifest that the risk signature established by 6 SE-lncRNAs may be used as an indicator to evaluate the clinical outcome of glioma patients.

Subsequently, the relationship between clinical information and our risk signature was investigated. Compared to patients in the low-risk group, the high-risk group presented more GBM phenotype, older age, death status, and cluster 3 in both CGGA (Fig. 3E) and TCGA (Additional file 2: Fig. S2C) datasets. In addition, recurrent gliomas were more likely to occur in the high-risk group of the training set (Fig. 3E). We further compared the differences in patients’ risk scores classified by various clinical characteristics. Results revealed that glioma patients with GBM phenotype, more than 60 years of age, dead status, or in cluster 3 had higher risk scores in CGGA (Fig. 3F–I) and TCGA (Additional file 2: Fig. S2D–G) datasets. Moreover, high-risk scores were also associated with unmethylated MGMT promoter, 1p19q non-code, recurrent glioma, and IDH wild-type genotype in the training set (Fig. 3J–M). Figure 3N and Additional file 2: Fig. S2H further showed the distribution of glioma patients in our model in two datasets.

Finally, we performed univariate Cox regression analysis on four clinical factors: age, gender, grade, and risk score. The p-value indicated that age, grade, and risk score were all related to the OS of glioma patients in the training (Fig. 3O) and validation (Additional file 2: Fig. S2I) sets. Further multivariate Cox regression analysis was conducted by integrating these four factors. The p-value suggested that the risk score could be an independent prognostic factor for glioma patients in both CGGA (Fig. 3P) and TCGA (Additional file 2: Fig. S2J) databases. In conclusion, we established a 6 SE-lncRNAs risk signature that could predict glioma patients’ survival.

### Functional analysis

**Fig. 4:**
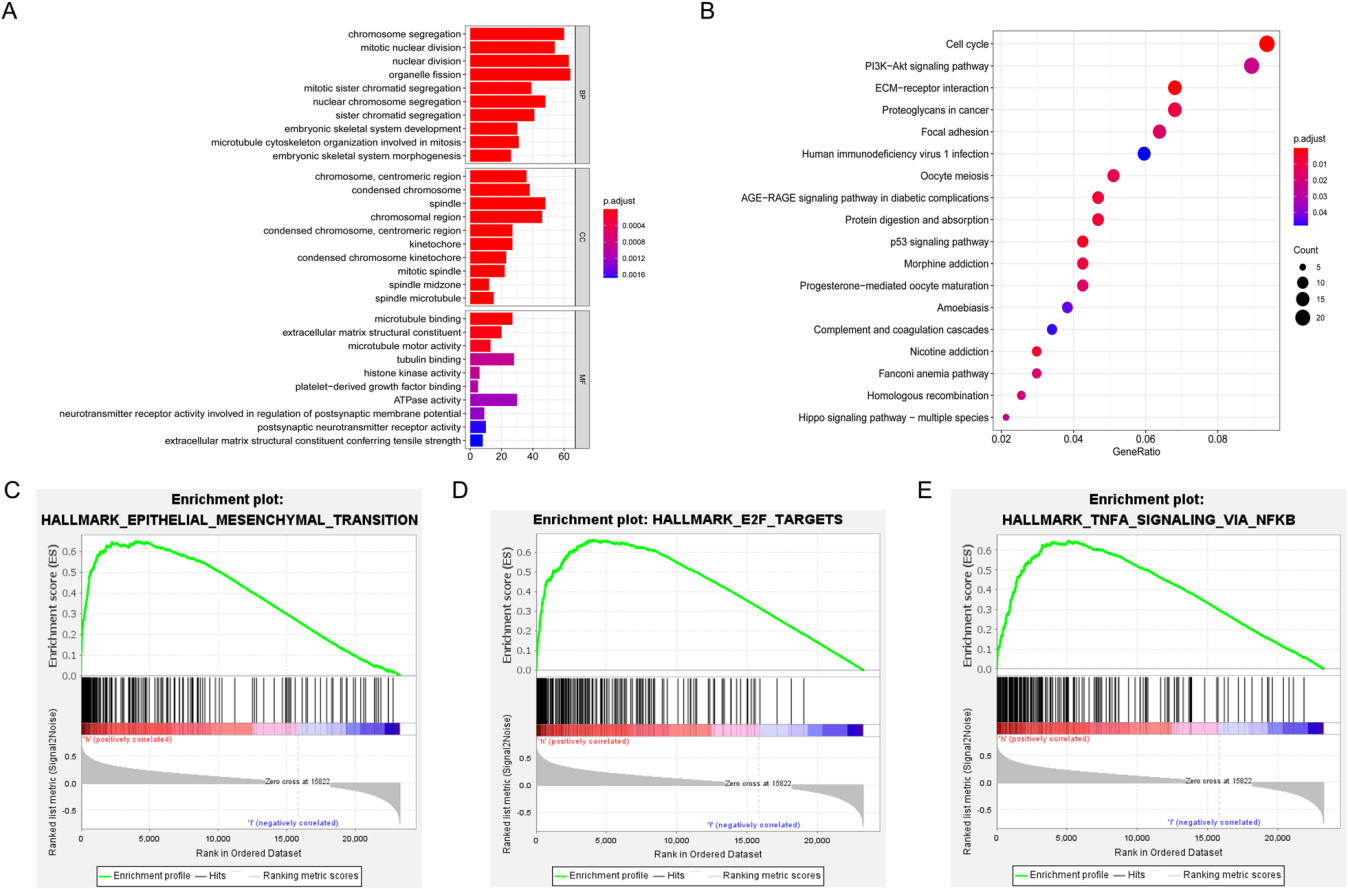
Functional analysis of the risk signature. **A** GO analysis of 6 SE-lncRNAs. **B** KEGG analysis of 6 SE-lncRNAs. **C**–**E** GSEA showed that the high-risk group enriched EMT, E2F targets, and the inflammatory pathway

To explore the potentially altered functional characteristics related to our SE-lncRNA signature, we conducted GO and KEGG analyses on the 6 selected SE-lncRNAs to study underlying biological processes related to glioma. GO analysis exhibited that 6 SE-lncRNAs were primarily associated with the cell cycle, including organelle fission, nuclear division, and chromosome segregation (Fig. 4A). KEGG analysis proved that these lncRNAs were principally correlated to cell cycle, phosphatidylinositol 3-kinase (PI3K)-Akt signaling pathway, the extracellular matrix (ECM)-receptor interaction, proteoglycans in cancer, and focal adhesion (Fig. 4B). Additionally, according to GSEA results, the high-risk group showed close association with EMT, E2F targets, and tumor necrosis factor α (TNFα) signaling via nuclear factor-κB (NFκB) (Fig. 4C–E). These results reveal the potential biological behavior and mechanism of 6 SE-lncRNAs affecting the prognosis of glioma.

### Immune characterization of two risk groups

**Fig. 5:**
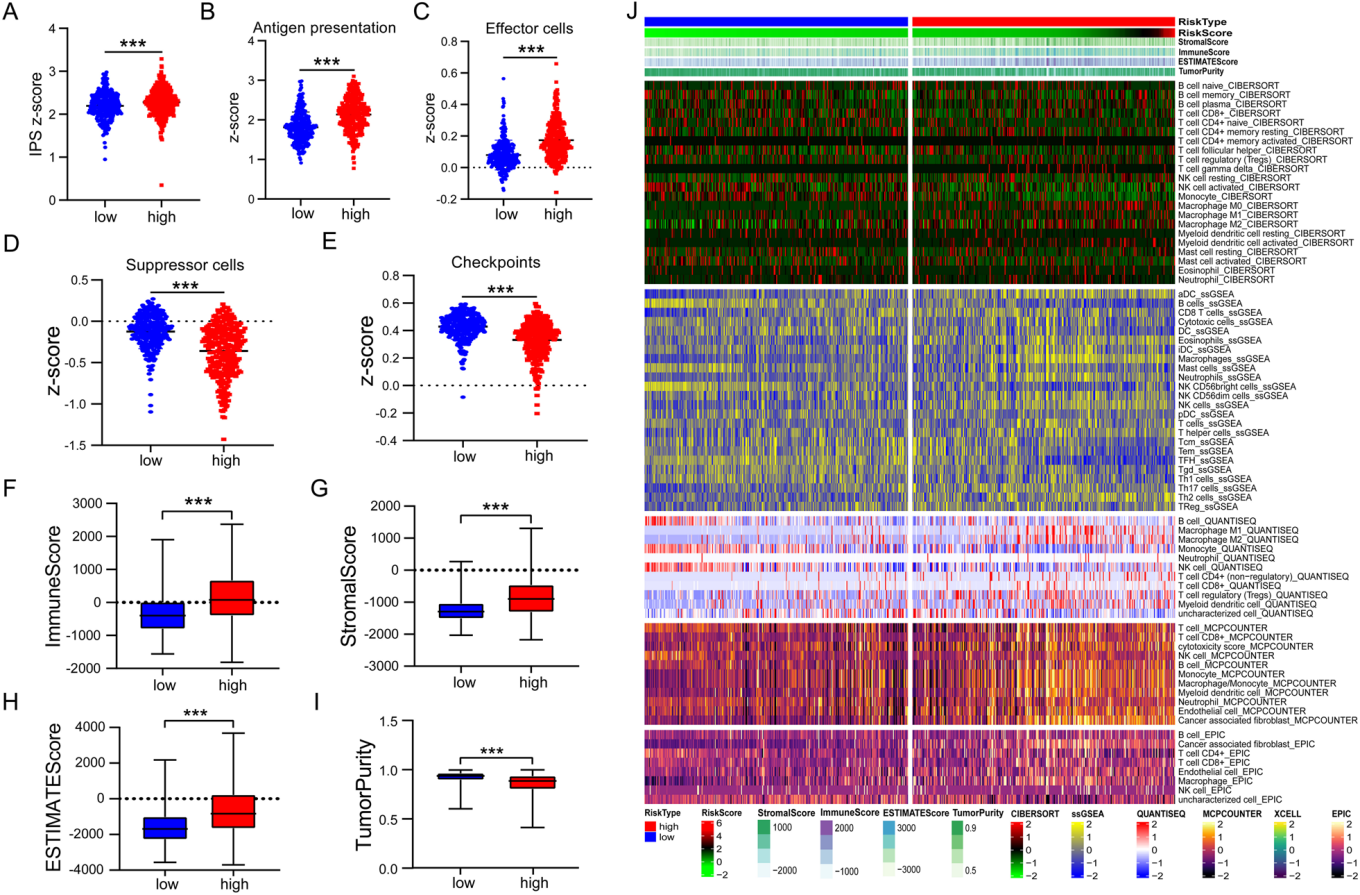
Estimating IPS scores, tumor purity, and TIIC components. **A**–**E** Boxplot showing differences of aggregated IPS z-score (**A**), antigen presentation (**B**), effector cells (**C**), suppressor cells (**D**), and checkpoint scores (**E**) between two risk groups. **F**–**I** A significant difference was shown in the immune score (**F**), stromal score (**G**), ESTIMATE score (H), and tumor purity (I) between the two risk groups. **J** Heatmap for the immune score, stromal score, ESTIMATE score and tumor purity, and immune cell infiltrates based on CIBERSORT, ssGSEA, QUANTISEQ, MCPCOUNTER, and EPIC algorithms in two risk groups. ***p < 0.001

The IPS score serves as a general indicator to evaluate the activation of the sample’s immune system. In our risk signature, the high-risk group presented a higher IPS z-score than the low-risk group, which suggested a different immune microenvironment modulation between the two groups (Fig. 5A). Specifically, the scores of antigen presentation and effector cells were higher in the high-risk group, while the scores for suppressor cells were lower (Fig. 5B–D). In addition, there were differences in the immune checkpoint category between the two risk groups (Fig. 5E). Next, we utilized the “ESTIMATE” R package to evaluate each glioma sample’s stromal and immune cell infiltration. As we can see, the high-risk group presented a higher immune score, which is consistent with IPS results (Fig. 5F). A higher overall abundance of stromal cells and ESTIMATE score but lower tumor purity was then observed in the high-risk group (Fig. 5G–I). We further explore this issue in more detail. Figure 5J shows the heatmap of immune infiltration according to CIBERSORT, ssGSEA, QUANTISEQ, MCPCOUNTER, and EPIC algorithms. Consistent results showed that the low-risk group presented higher abundances of natural killer cells (NK), T follicular helper cells (Tfh), and Th1 cells, while macrophage, regulatory T cells (Tregs), M2 macrophage, Cancer-associated fibroblasts (CAFs), neutrophils, dendritic cells (DCs), and CD8 + T cells were more common in high-risk groups.

### Correlation between prognostic signature of 6 SE-LncRNAs and response to immuno- and chemotherapies

**Fig. 6:**
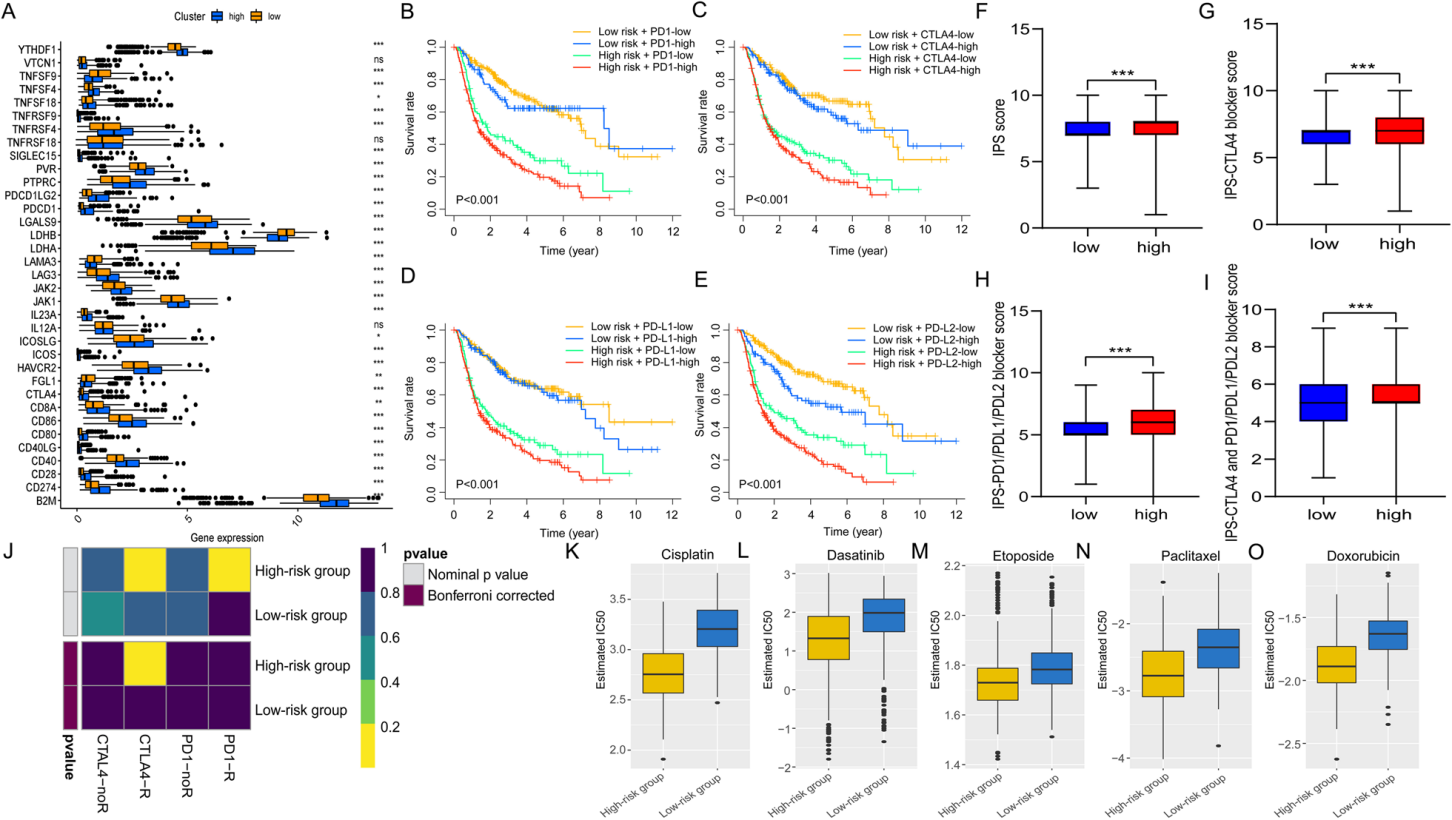
Differential expression of immune checkpoints and response to ICIs and chemotherapy between two risk groups. **A** Box plot presenting the expression of 35 immune checkpoints in low- and high-risk groups. **B**–**E** Kaplan–Meier OS curves among four patient groups stratified by the 6 SE-lncRNAs risk signature and PD1 (**B**), CTLA4 (**C**), PD-L1 (**D**), and PD-L2 (**E**). **F**–**I** The relationship between IPS and the 6 SE-lncRNA signature of glioma patients. Distribution plot of IPS score (**F**), IPS-CTLA4 blocker score (**G**), IPS-PD1/PDL1/PDL2 blocker score (**H**), and IPS-CTLA4 and PD1/PDL1/PDL2 blocker score (**I**) were shown. (**J**) Submap analysis demonstrated that the high-risk group was more sensitive to anti-CTLA-4 therapy (*P* < 0.05). **K–O** The SE-lncRNA signature could serve as a potential indicator to evaluate the sensitivity to cisplatin (**K**), dasatinib (**L**), etoposide (**M**), paclitaxel (**N**), and doxorubicin (**O**). *p < 0.05, **p < 0.01, ***p < 0.001 and ns indicates no significance

Given that the prognostic model correlated with tumor immunity, we further investigated the relationship between our prognostic model and immune checkpoint genes. Figure 6A showed the expression differences of 35 immune checkpoint genes between low- and high-risk groups, and there were 28 (e.g., PD-1, PD-L1, PD-L2, and CTLA4) of 35 genes observably up-regulated in high-risk patients (Fig. 6A). Further analysis revealed that the risk score was positively correlated with the expression of common immune checkpoint-related genes (Additional file 3: Fig. S3A–F). Besides, glioma patients stratified based on risk score and immune checkpoints genes expression performed significant survival differences. Patients in the low-risk group had better prognoses regardless of the highly or lowly expressed immune checkpoints (PD1, CTLA4, PD-L1, and PD-L2) (Fig. 6B–E). Recent reports have highlighted the role of the IPS based on tumor immunogenicity in predicting the immunotherapy response to ICI therapy. A higher IPS score indicates a better immunotherapy response [25, 34]. We further evaluated the efficacy of immunotherapeutics based on our risk signature. Three subtypes of IPS values (IPS-CTLA4 positive, IPS-PD-1/PD-L1/PD-L2 positive, and IPS-CTLA4/PD-1/PD-L1/PD-L2 positive) were served to evaluate responses of glioma patients to immunotherapy. Results showed that the high-risk group presented higher blocker scores in the above three subtypes (all *p* < 0.001; Fig. 6F–I), suggesting that patients in the high-risk group are inclined to benefit from immunotherapy. Subsequently, we further verified this result by performing the SubMap analysis. Results revealed that the expression profiles of patients in the high-risk group were related to those in the CTLA4 response group (*p* < 0.05), Which indicated that high-risk patients might respond better to anti-CTLA4 therapy, and also supports the results of IPS (Fig. 6J).

Considering that chemotherapy is a common treatment for glioma, we evaluated the treatment response of two risk groups to several common chemotherapeutic drugs, including cisplatin, dasatinib, etoposide, paclitaxel, and doxorubicin. The IC50 value of each glioma patient was calculated according to a predictive model of the corresponding drugs above. We observed a striking difference in the results of the two risk groups. The IC50 value belonging to the high-risk group was all lower, which means that high-risk patients could be more sensitive to commonly used chemotherapy drugs (all *p* < 0.001) (Fig. 6K–O).

### *BRD4 may regulate the expression of SE-LINC00945 *via* MED1*

**Fig. 7:**
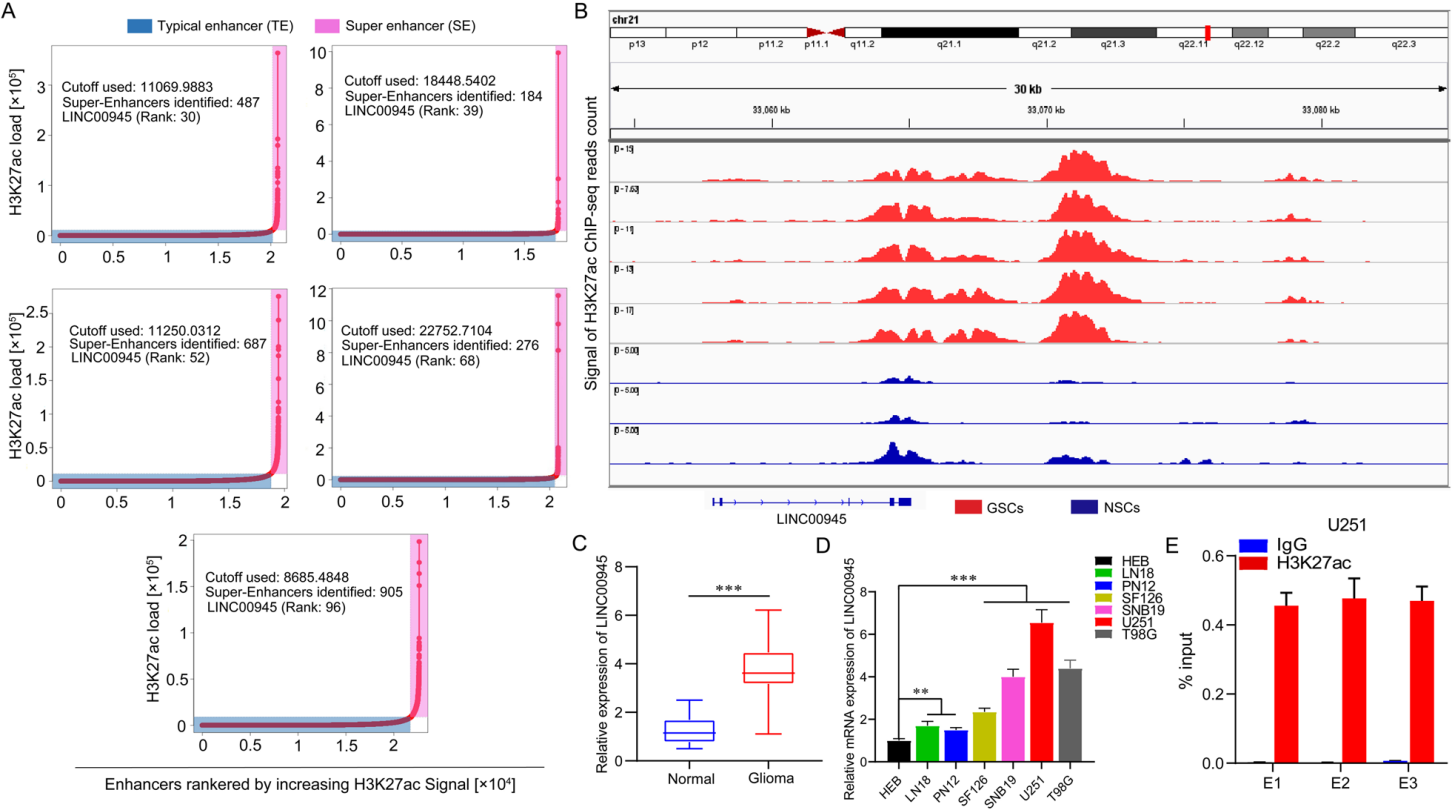
Epigenomic enhancer profiling defined LINC00945 as a SE-lncRNA. **A** Enhancer regions of GSCs. Enhancers were ranked by their H3K27ac ChIP-seq signals. Typical enhancers under the curve's inflection were marked in the blue box, while SEs above the inflection point were indicated in the red box. The number of SEs and rank of SE-LINC00945 were presented for each sample. **B** ChIP-seq profiles of H3K27ac in GSCs and NSCs. **C** Relative LINC00945 expression in glioma and normal brain tissues from our cohort. **D** Relative expression of LINC00945 in HEB, LN18, PN12, SF126, SNB19, U251 and T98G cells. **E** H3K27ac ChIP followed by RT-qPCR identified the SE of LINC00945. The co-immunoprecipitated DNA was amplified by PCR utilizing indicated primer. **p < 0.01, ***p < 0.001. The independent biological experiments were repeated at least three times

Subsequently, we obtained the expression of 6 SE-lncRNAs in multiple tumor types and paired normal tissues via the GEPIA2 website (https://gepia2.cancer-pku.cn). The results revealed that the high expression of LINC00945 was specific in glioma (Additional file 4: Fig. S4). To further explore the expression mechanism of LINC00945 in glioma, we selected five glioma patient-derived GSCs to profile the landscape of active enhancers, based on the fact that the rank ordering of SE-LINC00945 in GSCs ChIP-seq data of these five patients was in the top 5 of 43 glioma patients (Fig. 7A). In addition, H3K27ac ChIP-seq data generated in GSCs identified the SE of LINC00945 (Fig. 7B) and SEs of LHFPL3-AS1, HOXA-AS2, HOXA-AS3, SLC26A4-AS1, and HOTAIRM1 were also shown (Additional file 5: Fig. S5A-E). To investigate the role of LINC00945 in glioma, we detected LINC00945 expression in human glioma and normal brain tissue from our cohort. Figure 7C showed that LINC00945 expression was significantly up-regulated in glioma tissues compared to normal brain tissues. In addition, the expression levels of LINC00945 in normal human astrocyte (HEB), human glioma cell lines (LN18, SF126, SNB19, U251, and T98G), and human GBM cell PN12 derived from surgical specimens were measured. Compared with HEB, LINC00945 in various glioma cells showed highly expressed (Fig. 7D). We further verified the SE of LINC00945 by ChIP assay (Fig. 7E). Research indicated that the combination of ROSE with H3K27ac ChIP-seq can identify SEs. Besides, several cofactors (MED1), chromatin regulators (BRD4), and signaling factors (CDK7) can also be utilized for SE identification [35, 36].

**Fig. 8:**
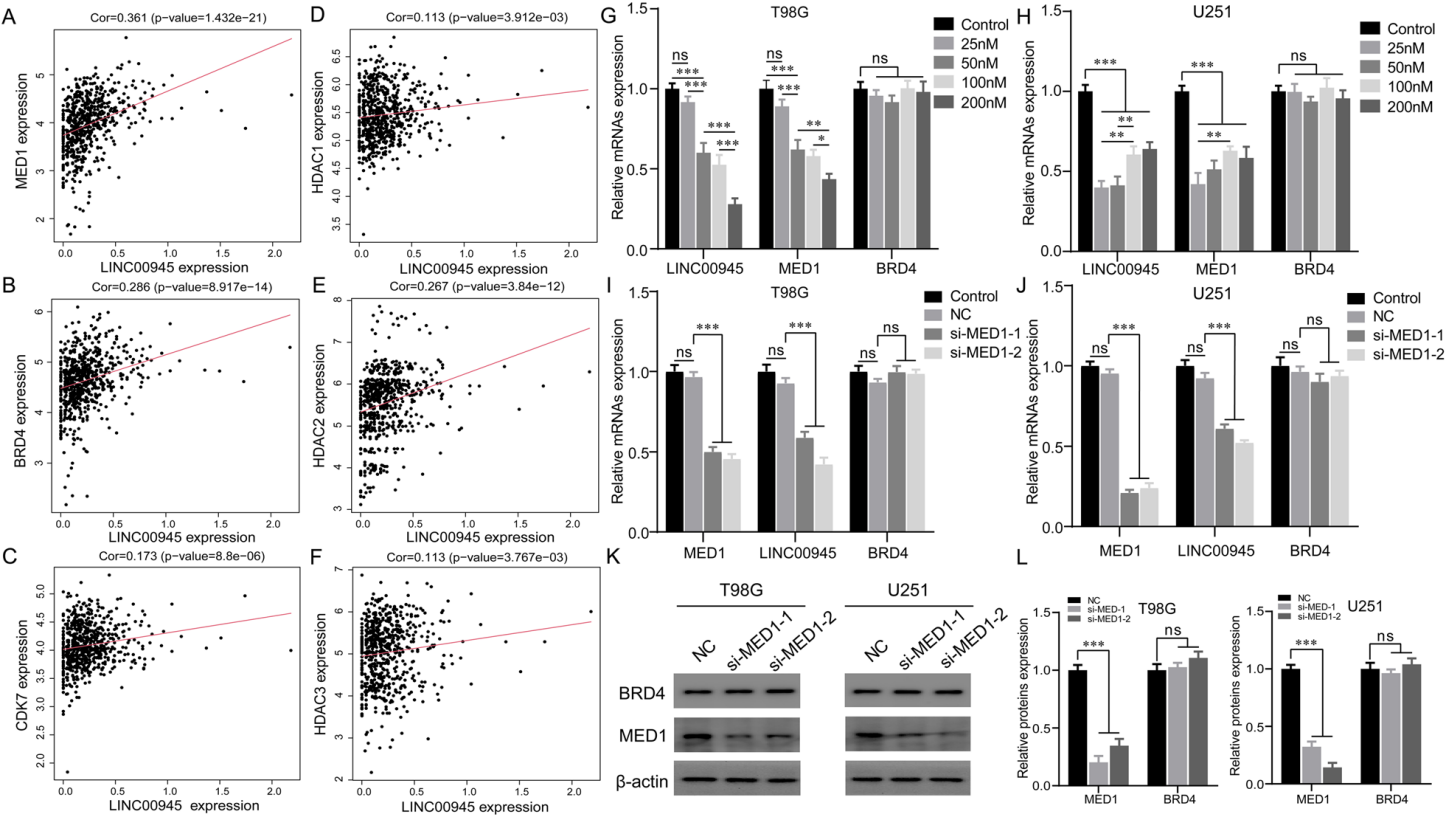
BRD4 promoted the transcription of LINC00945 via MED1. **A**–**F** The correlation of LINC00945 with MED1 (**A**), BRD4 (**B**), CDK7 (**C**), HDAC1 (**D**), HDAC2 (**E**), and HDAC3 (**F**) in the TCGA database, respectively. (G, H) The BRD4 inhibitor lessened the expression of LINC00945 and MED1 in both T98G (**G**) and U251 (**H**) cells. **I**, **J** The expression of LINC00945 decreased, while the mRNA expression of BRD4 was not affected after knocking down the expression of MED1 by siRNA. **K**, **L** The protein expression levels of BRD4 and MED1 were detected by western blot when treated with the siRNA of MED1. *p < 0.05, **p < 0.01, ***p < 0.001, and ns indicates no significance. The independent biological experiments were repeated at least three times

We then analyzed the correlation of LINC00945 with MED1, BRD4, CDK7, and Histone deacetylases (HDACs) in TCGA (Fig. 8A–F) and CGGA (Additional file 3: Fig. S3G–L) datasets. The results showed that MED1 and BRD4 had the highest positive correlation with LINC00945. To explore whether and how MED1 and BRD4 affected LINC00945 expression at the transcriptional level, T98G and U251 glioma cells were treated with JQ1 in indicated concentration for 48 h. JQ1, a BRD4 inhibitor, selectively interacts with the BD1 and BD2 domains of BRD4 and disrupts the interaction of BRD4 protein with acetylated lysine [37]. Results indicated that JQ1 inhibited the expression of LINC00945 and MED1 in both T98G and U251 cells without affecting the mRNA expression of BRD4 (Fig. 8G, H). However, when we knocked down MED1 by siRNA, MED1 and LINC00945 decreased, but the mRNA expression of BRD4 had no significant change (Fig. 8I, J). We further examined the changes of BRD4 at the protein level, and the results also indicated that the expression of BRD4 was not affected after MED1 knockdown (Fig. 8K, L). These suggested that BRD4 may regulate the expression of LINC00945 via MED1.

### *LINC00945 facilitates glioma cell proliferation *in vitro* and tumor growth *in vivo

**Fig. 9:**
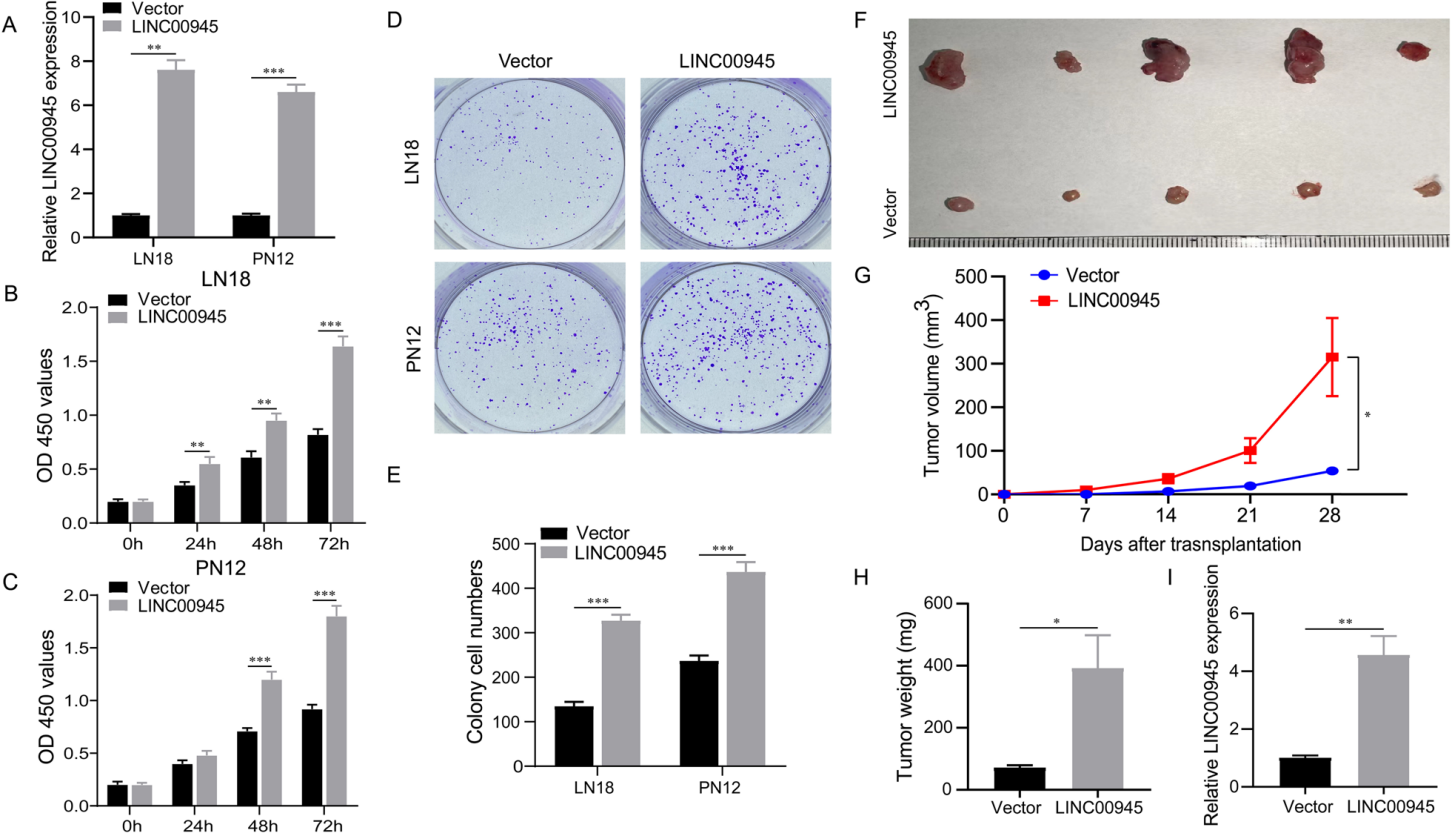
LINC00945 promotes glioma proliferation in vitro and in vivo. **A** The overexpression efficiency of LINC00945 was detected by qRT-PCR. **B**, **C** CCK8 experiments suggested that overexpression of LINC00945 promoted the proliferative capacity of LN18 (**B**) and PN12 cells (**C**). **D**, **E** Colony formation assay manifested LINC00945 increased LN18 and PN12 cells tumorigenicity. **F**–**H** The mouse xenograft model indicated that LINC00945 facilitated tumor growth in vivo. Images of tumor (**F**), volume growth curve (**G**), and tumor weight (H) were shown. (**I**) The differential expression of LINC00945 in xenograft tumor samples was detected by conducting RT-qPCR. *p < 0.05, **p < 0.01, ***p < 0.001. The independent biological experiments in vitro were repeated at least three times

We interfered with endogenous LINC00945 expression by LINC00945-overexpressing plasmid to elucidate the biological role of LINC00945 in glioma cells in vitro. RT-qPCR analysis confirmed the transfection efficiency (Fig. 9A). In the CCK-8 assay, overexpression of LINC00945 resulted in a marked proliferation increase (Fig. 9B, C). The colony-forming assay showed that overexpression of LINC00945 increased the colony-forming ability of both LN18 and PN12 glioma cells (Fig. 9D, E). We then evaluate the functional roles of LINC00945 in vivo xenograft models. PN12 cells with stable LINC00945 overexpression were constructed using lentivirus vectors and inoculated into nude mice. As illustrated in Fig. 9F and G, the size of tumor xenografts in the LINC00945 overexpression group was obviously bigger compared to the vector group, and tumor weight in the LINC00945 overexpression group is also heavier (Fig. 9H). In addition, expression levels of LINC00945 in nude mice tumor tissues were significantly increased in the LINC00945 overexpression group than in the vector group (Fig.  9I). These data indicated that LINC00945 promotes tumor proliferation in vivo and in vitro.

### LINC00945 promotes glioma cell EMT, migration, and invasion potential

**Fig. 10:**
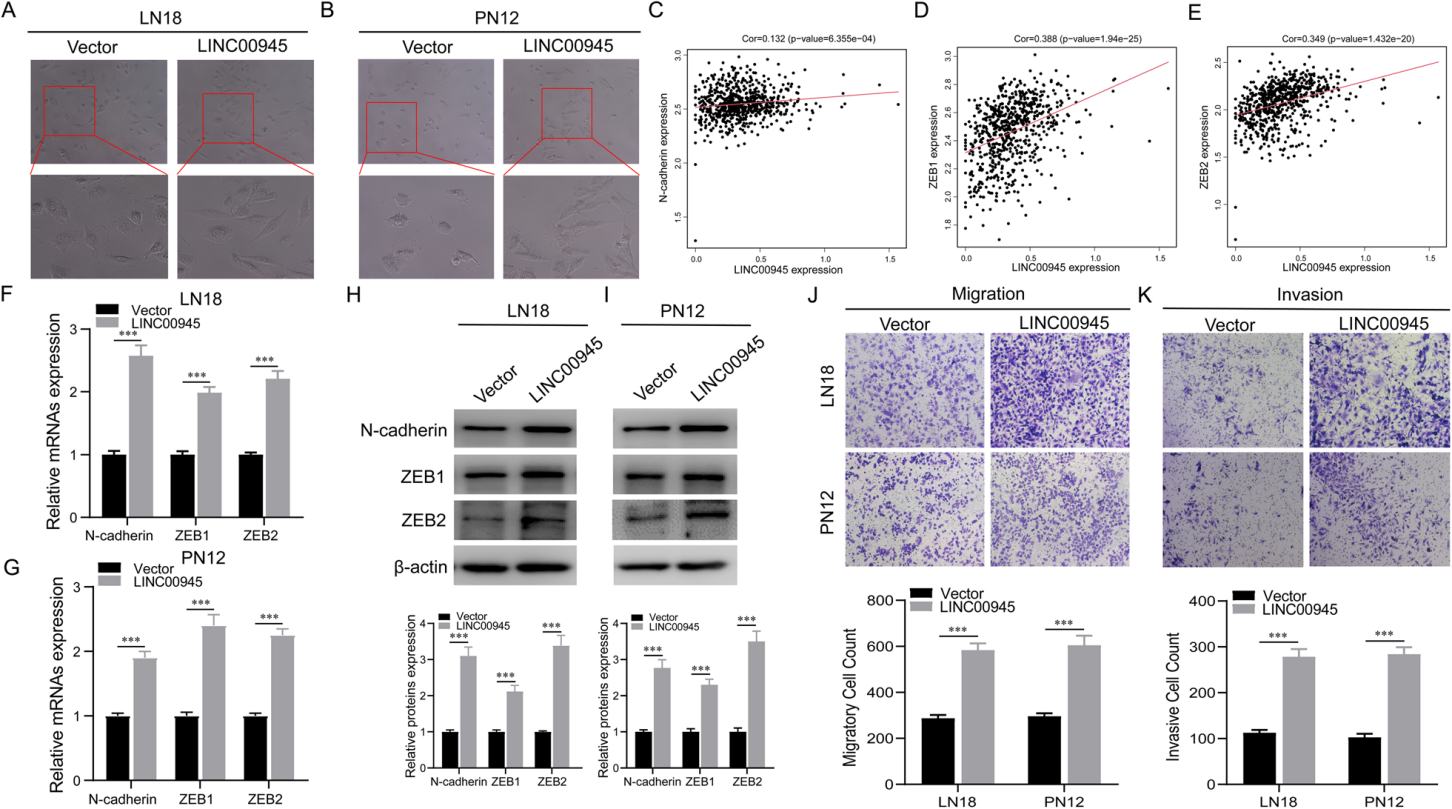
LINC00945 promotes EMT, migration, and invasion of glioma cells. **A**, **B** Morphological changes were perceived in both LN18 (**A**) and PN12 (**B**) cells after culturing for three weeks following overexpression LINC00945. **C**–**E** The expression of LINC00945 was positively correlated with the expression of N-cadherin (**C**), ZEB1 (**D**), and ZEB2 (**E**) in the TCGA database. **F**, **G** The mRNA expression levels of EMT-related genes (N-cadherin, ZEB1, and ZEB2) in LN18 (**F**) and PN12 (**G**) glioma cells transfected with LINC00945 and vector plasmids were examined by qRT-PCR. **H**, **I** Western blot examined the different expression levels of N-cadherin, ZEB1, and ZEB2 proteins in the LINC00945 overexpression and vector groups. **J**, **K** Transwell experiments revealed that overexpression of LINC00945 promoted LN18 and PN12 glioma cell migration (**J**) and invasion (**K**). ***p < 0.001. The independent biological experiments were repeated at least three times

Figure 10A and B show that more LN18 and PN12 cells exhibited spindle-shaped morphology meaning a more mesenchymal cell-like morphology after overexpression of LINC00945 and culturing for three weeks. Specifically, the proportion of cells with spindle-shaped morphology in vector groups of LN18 and PN12 cells was about 20% and 15%, respectively, compared with approximately 62% and 54% in the overexpressed LINC00945 groups. Correlation analyses further showed that the expression of LINC00945 was positively correlated with the expression of N-cadherin, ZEB1, and ZEB2 in both TCGA (Fig. 10C–E) and CGGA (Additional file 3: Fig. S3M–O) datasets. Consistent with this result, overexpression of LINC00945 led to significantly increased protein and mRNA expression levels of N-cadherin, ZEB1, and ZEB2 in LN18 and PN12 glioma cells (Fig. 10F–I). In transwell migration and invasion assays, we observed increased migratory and invasive glioma cells in the LINC00945 overexpression group (Fig. 10J, K). These findings suggest that LINC00945 contributes to glioma EMT, migration, and invasion.

## Discussion

Gliomas, the most common invasive primary malignant brain tumor of the CNS, exhibit poor prognosis [38]. SE-lncRNA is a crucial regulator of glioma. We have reported that SE-lncRNA TMEM44-AS1 promoted glioma progression by forming a positive feedback loop with Myc [33]. Some other SE-lncRNAs, including CCAT1, LINC00152, and NEAT1, may also facilitate the malignant phenotype of gliomas [39]. Recently, researchers have developed some lncRNA signatures that have shown predictive effects in the prognosis and treatment of glioma patients, including the pyroptosis-related lncRNA signature [40] and immune-related lncRNA signature [41]. Herein, we explored the predictive value of SE-lncRNAs in glioma prognosis and treatment.

In this work, we first identified 6 SE-lncRNAs by conducting a multi-omic analysis of RNA-seq and ChIP-seq data. Then, two datasets (CGGA and TCGA) were utilized to assess the prognostic value of 6 SE-lncRNAs in glioma patients. Based on the expression profiles of 6 SE-lncRNAs, we identified three glioma subgroups with different prognoses and clinicopathological characteristics via applying consensus clustering analysis. Similarly, Zhou et al. utilized immune-related genes to identify three glioma subgroups, which proved to be valid prognostic factors [42]. Zheng et al. also identified three glioma subgroups based on endoplasmic reticulum stress-associated lncRNAs [43]. Then, a prognostic risk signature with 6 SE-lncRNAs was established by performing the Lasso regression analysis. Patients in the high-risk group had a worse prognosis, showing more GBM subtypes, advanced age, dead status, and recurrent glioma. In addition, our molecular typing also correlates with the prognostic model. The cluster 3 subgroup with the worst prognosis was almost distributed in the high-risk group, while the cluster 1 subgroup with a better prognosis was almost in the low-risk group. The methylation of MGMT promoter is a vital reference factor for the clinical treatment of glioma patients, which indicates that glioma patients have a better therapeutic response to the first-line chemotherapy drug temozolomide [44]. In addition, 1p19q codeletion and IDH mutation have been reported to be associated with favorable prognosis in glioma patients [45]. In our risk model, glioma patients with MGMT promoter methylation, 1p19q codeletion, and IDH mutation presented a relatively lower risk score, suggesting a better prognosis. Research shows that a single model integrating multiple biomarkers performs better predictive effects than a single biomarker [46]. Consistently, univariate and multivariate analyses confirmed that our risk score could be a valuable indicator for evaluating the prognosis of glioma patients.

In consideration of the vital role of our risk signature in assessing glioma prognosis, we further focused on underlying mechanisms. GO and KEGG analyses revealed that SE-lncRNAs closely correlated with cell cycle, PI3K-Akt signaling pathway, ECM-receptor interaction, proteoglycans in cancer, and focal adhesion. Dysregulation of the cell cycle is a crucial mechanism for the infinite proliferation and metastasis of malignant glioma cells [47]. PI3K-AKT signaling pathway is an essential intracellular signal pathway that is directly involved in regulating glioma cell proliferation, EMT, migration, invasion, and immune escape [48–51]. As a complex extracellular macromolecular network, ECM controls multiple cellular activities, such as adhesion, migration, and proliferation [52]. In addition, the intricate immunosuppressive networks formed by stromal cells, inflammatory cells, vasculature, ECM, and their secreted cytokines in the TME, play a pivotal role in tumor immune escape [53]. Proteoglycans, key molecular effectors of cell surface and pericellular microenvironments, perform multiple functions in cancer, including regulating tumor cell growth, tumor microenvironment, and metastasis [54]. Focal adhesion has been reported to play a vital role in cancer cell migration and metastasis by linking the actin cytoskeleton to the ECM, allowing the cell to generate traction [55, 56]. The GSEA suggested that EMT, E2F targets, and inflammation-related pathways were markedly enriched in the high-risk group. EMT is thought to be a typical pattern of malignant epithelial tumor progression, inducing locally invasive and metastatic tumor subtypes [57]. As vital drivers of tumor growth, E2F transcription factors participate in various tumor advances, including cell cycle, invasion, and DNA damage response [58]. TNF‐α, an inflammatory factor, is considered to be remarkably related to the malignant progression of gliomas, and NFκB sustainedly activated by TNF-α results in invasion and metastasis of tumor cells [59]. Consequently, we speculated that 6 SE-lncRNAs might mainly affect the biological behaviors of glioma cells, including proliferation, EMT, migration, and invasion, and participate in the regulation of TME.

The formation of a tumor is usually accompanied by the formation of a tumor bed, changes in surrounding connective tissue and matrix, and, ultimately, a microenvironment suitable for the survival of tumor cells [60]. A better understanding of the TME will contribute to developing new ways to improve the TME, thereby enhancing the effect of immunotherapy, which also lays a solid foundation for the application of immunotherapy combined with other therapy in the future. Abundant glioma-related nontumor cells, represented by stromal and immune cells in TME, play a crucial role in glioma progression [61, 62]. Specifically, glioma-related stromal cells, such as astrocytes, endothelial cells, and mesenchymal cells, play an essential role in tumorigenesis, angiogenesis, and invasion [63]. TIICs can induce tumor immunosuppression and immune evasion, and different immune cells play different roles in gliomas [64]. Our results presented a higher z-score of IPS in the high-risk group, indicating higher immunogenicity [25]. Meanwhile, the ESTIMATE algorithm uncovered that our high-risk group had higher stromal and immune scores. Tumor purity refers to the percentage of cancer cells in a tumor sample, so patients in the high-risk group presented lower tumor purity. The report indicated that low tumor purity is associated with a poor prognosis of glioma [65], and our results support this conclusion.

Subsequently, we utilized multiple algorithms to compare differences in the distribution of TIICs between the two risk groups. NK cells, Tfh cells, and Th1 cells, exhibiting anti-tumoral functions, were more common in the low-risk group [66–68], while the high-risk group presented higher enrichment of macrophage, Tregs, M2 macrophage, CAFs, neutrophils, DCs, and CD8 + T cells. Previous studies have shown that tumor-associated macrophages (TAMs) occupied 30–40% of the cellular component in GBM, identified as the promoter of tumor progression due to the immunosuppressive function [69]. TAMs of the M2 phenotype are closely associated with tumor cell proliferation and unfavorable clinical prognosis in GBM patients [70]. In addition, tumors secrete the chemokine CCL28, selectively attracting Tregs and increasing their functions, which can inhibit effector T cells and DCs from exerting immunosuppressive effects [71]. Several studies have indicated that the proportion of Tregs infiltration in glioma increased with the malignant degree of tumor [72, 73]. CAFs, the main cellular component of cancer stroma, play a key role in inducing EMTs, maintaining tumor stem cell pools, regulating tumor immunity, promoting tumor immune escape, and resisting immunotherapy [74–76]. The infiltration level of neutrophils was positively correlated with glioma grade. The immunosuppression and angiogenesis induced by neutrophils could promote the progression of glioma [77, 78]. As professional antigen-presenting cells, DCs stimulate the anti-tumor effect of CD8 + T cells by presenting tumor antigens; however, M2 macrophage, Tregs, and CAFs could form immune barriers against CD8 + T cell-mediated anti-tumor immune responses [79, 80]. Thus, although CD8 + T cells in the high-risk group are more abundant, these cells may not perform their function well. In a word, the high-risk group tends to exhibit an immunosuppressive microenvironment responsible for poor prognosis.

In recent years, immunotherapy represented by anti-PD1 therapy has shown promising therapeutic potential in partial preclinical research of glioma [81, 82]; however, most clinical trials of immunotherapy have failed to achieve the expected treatment efficacy [83], and the clinical benefit of ICIs therapy to glioma patients is highly heterogeneous. Thus, screening suitable patients is a critical factor in improving the therapeutic efficacy of ICIs. Based on our risk signature, we further explored the correlation between the risk score and expression levels of crucial immune checkpoints. The high-risk group presented a higher expression of PD1 (PDCD1), CTLA4, PD-L1 (CD274), and PD-L2 (PDCD1LG2), and our risk signature could also distinguish glioma patients with similar expression levels of immune checkpoints. PD-1 was reported to play an essential role in inhibiting immune responses and promoting self-tolerance through regulating the activity of T cells and blocking the apoptosis of Tregs. Tumor cells usually up-regulate the expression of PD-1 ligands (either PD-L1 or PD-L2) to suppress the anti-tumor immune response [84, 85]. CTLA4, a surface receptor of Tregs, can suppress the activation of effector T cells [86]. Moreover, it is generally believed that patients with high expression of immune checkpoint genes, which could theoretically provide therapeutic targets, are more likely to benefit from ICIs treatment [87–89]. Some immune cells are also associated with ICIs treatment. Accumulating evidence indicates that DCs improve responsiveness to anti-PD-1 immunotherapy, and the efficacy of ICIs is dependent on a high density of preexistent CD8 + T cells [90, 91]. Thus, we speculated that patients in the high-risk group are more likely to benefit from ICIs. Results of IPS, a good predictor of the treatment response to ICIs [25], revealed that the high-risk group presented higher immune checkpoint blocker scores, which is consistent with our speculation. In addition, the submap analysis also revealed that the high-risk group was more suitable for anti-CTLA4 treatments. These results indicated that our SE-lncRNA signature is a potential model that can evaluate glioma patients more likely to respond to ICIs. We further studied the response of our risk signature to several common chemotherapy drugs utilizing the GDSC database, and results revealed that the high-risk group was overall more sensitive to commonly administered chemotherapies. In addition, chemotherapy has been widely explored as a suitable partner for anti-PD-1/PD-L1 and anti-CTLA-4 therapy based on the immunomodulatory effects of chemotherapy drugs. For example, in the clinical trial KEYNOTE-021 (phase 2), carboplatin and pemetrexed in combination with pembrolizumab (PD-1 inhibitor) significantly improved response rate and progression-free survival (PFS) in patients with advanced non-squamous non-small cell lung cancer [92]. Thus, Patients in the high-risk group may achieve a better therapeutic effect from the combination of immunotherapy and chemotherapy.

6 SE-lncRNAs are contained in our risk signature. Among them, five lncRNAs have been reported to be related to the malignant progression of tumors [93–97]. However, the role of LINC00945 has not been explored. In addition, the high expression of LINC00945 is specific in gliomas, and LINC00945 has the highest regression coefficient value in our prognostic model, indicating that it contributes the most to the model. Thus, we explored the potential expression mechanism and function of LINC00945 in gliomas. According to the report, the combination of the ROSE program with ChIP-seq targeting H3K27ac is widely used to identify SEs [35, 36]. In the present study, we identified the SE of LINC00945 by analyzing H3K27ac ChIP-seq data, which was further verified by performing the ChIP assay. As the second SEs marker after H3K27ac in various cell types, BRD4, a member of the bromodomain and extra terminal domain (BET) protein family, functions as a transcriptional regulator and epigenetic reader in cells that binds to acetylated lysine in histones [98]. Nishida et al. treated renal cancer cells with BET inhibitor JQ1 to reduce DRD4 binding and CXC chemokine transcription, thereby inhibiting renal cancer metastasis and neutrophil infiltration [99]. MED1 is a cofactor promoting transcription initiation and acts as a bridge between enhancers and promoters [100]. In addition, the occupancy of MED1 serves as concrete evidence for identifying enhancer elements [101]. A study from Richard Young’s group showed that transcriptional coactivators, BRD4 and MED1, form phase-separated condensates at SEs, which gathers the transcription machinery near the SEs and results in robust expression of SE-genes [102]. Herein, we preliminarily revealed that BRD4 might regulate the expression of LINC00945 via MED1. However, the exact mechanism needs to be further explored. Subsequently, based on the results of functional experiments and the mouse xenograft model, we verify that LINC00945 promotes proliferation, EMT, migration and invasion of glioma cells, and tumor growth in vivo, suggesting that LINC00945 may serve as a novel underlying therapeutic target for glioma.

## Conclusions

In conclusion, we identified three glioma subgroups with disparate prognostic and clinical features based on the screened 6 SE-lncRNAs and developed a 6 SE-lncRNAs expression-based risk signature with an ability to predict glioma prognosis and immuno- and chemotherapeutic response. Meanwhile, different immune characteristics between the two risk groups were presented. Furthermore, we explored the epigenetic activation mechanism of LINC00945 and its effect on glioma. In short, the present study has guidance value for clinicians to analyze prognosis and achieve individualized treatment for glioma patients.

## Supplementary Information

**Additional file 1**: **Fig. S1** 6 SE-lncRNAs classified glioma patients into 3 clusters in the TCGA dataset. **A** CDF of consensus clustering for k = 2 to 9. **B** A relative change in area under the CDF curve was shown. **C** The consensus clustering matrix revealed that patients were divided into 3 clusters (k = 3). **D** Survival difference of patients in cluster 1/2/3 subgroups. **E** Heatmap of clinicopathologic distribution and 6 SE-lncRNAs expression levels among cluster 1/2/3. **F**–**H** The clinicopathological features of grade (**F**), age (**G**), and survival status (**H**) in 3 subgroups. ***p < 0.001.

**Additional file 2**: **Fig. S2** Verification of risk model in TCGA dataset. **A** Kaplan-Meier OS curves for patients in two risk groups. **B** ROC curves presented the predictive efficiency of our risk model in the validation set. **C** The heatmap displayed the differential clinicopathological features and 6 SE-lncRNAs expression levels in two risk groups. **D**–**G** The WHO grade (**D**), cluster 1/2/3 subgroups (**E**), age (**F**), and fustat (**G**) stratified the validation set, and the distribution of risk scores was shown. **H** Distribution and survival differences of patients in two risk groups in the TCGA dataset. **I**, **J** Univariate (**I**) and multivariate (**J**) Cox analyses included age, gender, WHO grade, and risk score in the validation set. ***p < 0.001.

**Additional file 3**: **Fig. S3** The correlation analysis of risk score and immune checkpoint genes, and LINC00945 and super-enhancer related factors or EMT-related genes expression in CGGA dataset. **A**–**F** Correlation between risk scores and immune checkpoint genes, including PD1 (**A**), PD-L1 (**B**), PD-L2 (**C**), CD247 (**D**), CTLA4 (**E**), and IDO1 (**F**). **G–L** The correlation of LINC00945 with MED1 (**G**), BRD4 (**H**), CDK7 (**I**), HDAC1 (**J**), HDAC2 (**K**), and HDAC3 (**L**), respectively. **M–O** LINC00945 was positively related to the expression of N-cadherin (**M**), ZEB1 (**N**), and ZEB2 (**O**) in the CGGA database.

**Additional file 4**: **Fig. S4** The expression of LINC00945 (**A**), SLC26A4-AS1 (**B**), LHFPL3-AS1 (**C**), HOXA-AS2 (**D**), HOXA-AS3 (**E**), and HOTAIRM1 (**F**) in certain tumor type and normal tissue.

**Additional file 5**: **Fig. S5** H3K27ac ChIP-seq data identified SEs of LHFPL3-AS1 (**A**), HOXA-AS2 (**B**), HOXA-AS3 (**C**), SLC26A4-AS1 (**D**), and HOTAIRM1 (**E**) in GSCs.

**Additional file 6**: **Table S1** Clinic-pathological characteristics of 12 glioma patients. **Table S2** Primer pairs used for enhancer regions in ChIP-qPCR and performing RT-qPCR.

## Abbreviations

SE: Super-enhancer
lncRNA: Long non-coding RNA
SE-lncRNA: Super-enhancer-associated lncRNA
RNA-seq: RNA sequencing
ChIP-seq: ChIP sequencing
GSC: Glioma steam cell
NSC: Neural stem cell
GEO: Gene Expression Omnibus
CGGA: The Chinese Glioma Genome Atlas
TCGA: The Cancer Genome Atlas
CNS: Central nervous system
WHO: The World Health Organization
OS: Overall survival
PFS: Progression-free survival
GBM: Glioblastoma
TIL: Tumor-infiltrating lymphocyte
TIIC: Tumor-infiltrating immune cell
ICB: Immune checkpoint blockade
ICI: Immune checkpoint inhibitor
IPS: Immunophenoscore
ROC: Receiver operating characteristic
CDF: Cumulative distribution function
GO: Gene Ontology
KEGG: Kyoto Encyclopedia of Genes and Genomes
GSEA: Gene set enrichment analysis
ssGSEA: Single-sample gene set enrichment analysis
Tfh: T follicular helper cells
DCs: Dendritic cells
CAFs: Cancer-associated fibroblasts
TME: Tumor microenvironment
TIDE: Tumor immune dysfunction and exclusion
IC50: Half-maximal inhibitory concentration
GDSC: Genomics of Drug Sensitivity in Cancer
siRNA: Small interfering RNA
ROSE: Rank Ordering of Super-Enhancers
ChIP: Chromatin immunoprecipitation
RT-qPCR: Real-time quantitative PCR
CCK-8: Cell counting kit-8
ECM: Extracellular matrix
TNFα: Tumor necrosis factor α
NFκB: Nuclear factor-κB
HDAC: Histone deacetylase
TAM: Tumor-associated macrophage
Treg: Regulatory T cells
NK: Natural killer cells
BET: Bromodomain and extraterminal domain
EMT: Epithelial-mesenchymal transition

## References

[1] Reni M, Mazza E, Zanon S, Gatta G, Vecht CJ (2017). Central nervous system gliomas. Crit Rev Oncol Hematol.

[2] Louis DN, Perry A, Reifenberger G, von Deimling A, Figarella-Branger D, Cavenee WK (2016). The 2016 World Health Organization classification of tumors of the central nervous system: a summary. Acta Neuropathol.

[3] Tan AC, Ashley DM, Lopez GY, Malinzak M, Friedman HS, Khasraw M (2020). Management of glioblastoma: State of the art and future directions. CA Cancer J Clin.

[4] Kaymak I, Williams KS, Cantor JR, Jones RG (2021). Immunometabolic Interplay in the Tumor Microenvironment. Cancer Cell.

[5] Petitprez F, de Reynies A, Keung EZ, Chen TW, Sun CM, Calderaro J (2020). B cells are associated with survival and immunotherapy response in sarcoma. Nature.

[6] Salem D, Chelvanambi M, Storkus WJ, Fecek RJ (2021). Cutaneous melanoma: mutational status and potential links to tertiary lymphoid structure formation. Front Immunol.

[7] Sampson JH, Gunn MD, Fecci PE, Ashley DM (2020). Brain immunology and immunotherapy in brain tumours. Nat Rev Cancer.

[8] Ribas A, Wolchok JD (2018). Cancer immunotherapy using checkpoint blockade. Science.

[9] Postow MA, Chesney J, Pavlick AC, Robert C, Grossmann K, McDermott D (2015). Nivolumab and ipilimumab versus ipilimumab in untreated melanoma. N Engl J Med.

[10] Slater RL, Lai Y, Zhong Y, Li H, Meng Y, Moreno BH (2020). The cost effectiveness of pembrolizumab versus chemotherapy or atezolizumab as second-line therapy for advanced urothelial carcinoma in the United States. J Med Econ.

[11] Mansour MR, Abraham BJ, Anders L, Berezovskaya A, Gutierrez A, Durbin AD (2014). Oncogene regulation. An oncogenic super-enhancer formed through somatic mutation of a noncoding intergenic element. Science.

[12] Kalna V, Yang Y, Peghaire CR, Frudd K, Hannah R, Shah AV (2019). The transcription factor ERG regulates super-enhancers associated with an endothelial-specific gene expression program. Circ Res.

[13] Sanchez GJ, Richmond PA, Bunker EN, Karman SS, Azofeifa J, Garnett AT (2018). Genome-wide dose-dependent inhibition of histone deacetylases studies reveal their roles in enhancer remodeling and suppression of oncogenic super-enhancers. Nucleic Acids Res.

[14] Hnisz D, Abraham BJ, Lee TI, Lau A, Saint-Andre V, Sigova AA (2013). Super-enhancers in the control of cell identity and disease. Cell.

[15] Liu K, Gao L, Ma X, Huang JJ, Chen J, Zeng L (2020). Long non-coding RNAs regulate drug resistance in cancer. Mol Cancer.

[16] Huang Z, Zhou JK, Peng Y, He W, Huang C (2020). The role of long noncoding RNAs in hepatocellular carcinoma. Mol Cancer.

[17] McCabe EM, Rasmussen TP (2021). lncRNA involvement in cancer stem cell function and epithelial-mesenchymal transitions. Semin Cancer Biol.

[18] Peng L, Jiang B, Yuan X, Qiu Y, Peng J, Huang Y (2019). Super-enhancer-associated long noncoding RNA HCCL5 is activated by ZEB1 and promotes the malignancy of hepatocellular carcinoma. Cancer Res.

[19] Yan L, Chen H, Tang L, Jiang P, Yan F (2021). Super-enhancer-associated long noncoding RNA AC005592.2 promotes tumor progression by regulating OLFM4 in colorectal cancer. BMC Cancer.

[20] Lv W, Wang Y, Zhao C, Tan Y, Xiong M, Yi Y (2021). Identification and validation of m6A-related lncRNA signature as potential predictive biomarkers in breast cancer. Front Oncol.

[21] Yu W, Ma Y, Hou W, Wang F, Cheng W, Qiu F (2021). Identification of immune-related lncRNA prognostic signature and molecular subtypes for glioblastoma. Front Immunol.

[22] Li R, Qian J, Wang YY, Zhang JX, You YP (2014). Long noncoding RNA profiles reveal three molecular subtypes in glioma. CNS Neurosci Ther.

[23] Sauerbrei W, Royston P, Binder H (2007). Selection of important variables and determination of functional form for continuous predictors in multivariable model building. Stat Med.

[24] Yoshihara K, Shahmoradgoli M, Martinez E, Vegesna R, Kim H, Torres-Garcia W (2013). Inferring tumour purity and stromal and immune cell admixture from expression data. Nat Commun.

[25] Charoentong P, Finotello F, Angelova M, Mayer C, Efremova M, Rieder D (2017). Pan-cancer immunogenomic analyses reveal genotype-immunophenotype relationships and predictors of response to checkpoint blockade. Cell Rep.

[26] Newman AM, Liu CL, Green MR, Gentles AJ, Feng W, Xu Y (2015). Robust enumeration of cell subsets from tissue expression profiles. Nat Methods.

[27] Yi M, Nissley DV, McCormick F, Stephens RM (2020). ssGSEA score-based Ras dependency indexes derived from gene expression data reveal potential Ras addiction mechanisms with possible clinical implications. Sci Rep.

[28] Finotello F, Mayer C, Plattner C, Laschober G, Rieder D, Hackl H (2019). Molecular and pharmacological modulators of the tumor immune contexture revealed by deconvolution of RNA-seq data. Genome Med.

[29] Becht E, Giraldo NA, Lacroix L, Buttard B, Elarouci N, Petitprez F (2016). Estimating the population abundance of tissue-infiltrating immune and stromal cell populations using gene expression. Genome Biol.

[30] Racle J, de Jonge K, Baumgaertner P, Speiser DE, Gfeller D (2017). Simultaneous enumeration of cancer and immune cell types from bulk tumor gene expression data. Elife.

[31] Hoshida Y, Brunet JP, Tamayo P, Golub TR, Mesirov JP (2007). Subclass mapping: identifying common subtypes in independent disease data sets. PLoS ONE.

[32] Yang W, Soares J, Greninger P, Edelman EJ, Lightfoot H, Forbes S (2013). Genomics of Drug Sensitivity in Cancer (GDSC): a resource for therapeutic biomarker discovery in cancer cells. Nucleic Acids Res.

[33] Bian E, Chen X, Cheng L, Cheng M, Chen Z, Yue X (2021). Super-enhancer-associated TMEM44-AS1 aggravated glioma progression by forming a positive feedback loop with Myc. J Exp Clin Cancer Res.

[34] Xu F, Liu T, Zhou Z, Zou C, Xu S (2021). Comprehensive analyses identify APOBEC3A as a genomic instability-associated immune prognostic biomarker in ovarian cancer. Front Immunol.

[35] Wei Y, Zhang S, Shang S, Zhang B, Li S, Wang X (2016). SEA: a super-enhancer archive. Nucleic Acids Res.

[36] Bradner JE, Hnisz D, Young RA (2017). Transcriptional addiction in cancer. Cell.

[37] Filippakopoulos P, Qi J, Picaud S, Shen Y, Smith WB, Fedorov O (2010). Selective inhibition of BET bromodomains. Nature.

[38] Lim M, Xia Y, Bettegowda C, Weller M (2018). Current state of immunotherapy for glioblastoma. Nat Rev Clin Oncol.

[39] Cheng M, Zhang ZW, Ji XH, Xu Y, Bian E, Zhao B (2020). Super-enhancers: a new frontier for glioma treatment. Biochim Biophys Acta Rev Cancer.

[40] Tanzhu G, Li N, Li Z, Zhou R, Shen L (2022). Molecular subtypes and prognostic signature of pyroptosis-related lncRNAs in glioma patients. Front Oncol.

[41] Cao Y, Zhu H, Tan J, Yin W, Zhou Q, Xin Z (2021). Development of an immune-related LncRNA prognostic signature for glioma. Front Genet.

[42] Zhou Q, Yan X, Liu W, Yin W, Xu H, Cheng D (2020). Three immune-associated subtypes of diffuse glioma differ in immune infiltration, immune checkpoint molecules, and prognosis. Front Oncol.

[43] Zheng Y, Yue X, Fang C, Jia Z, Chen Y, Xie H (2022). A Novel defined endoplasmic reticulum stress-related lncRNA signature for prognosis prediction and immune therapy in glioma. Front Oncol.

[44] Hegi ME, Diserens A-C, Gorlia T, Hamou M-F, de Tribolet N, Weller M (2005). MGMT gene silencing and benefit from temozolomide in glioblastoma. N Engl J Med.

[45] Eckel-Passow JE, Lachance DH, Molinaro AM, Walsh KM, Decker PA, Sicotte H (2015). Glioma groups based on 1p/19q, IDH, and TERT promoter mutations in tumors. N Engl J Med.

[46] Zhang C, Jing LW, Li ZT, Chang ZW, Liu H, Zhang QM, et al. Identification of a prognostic 28-gene expression signature for gastric cancer with lymphatic metastasis. Biosci Rep. 2019;39(5). 10.1042/BSR20182179 PMC6499450 30971501

[47] Li X, Wang H (2021). Long non-coding RNA GABPB1-AS1 augments malignancy of glioma cells by sequestering microRNA-330 and reinforcing the ZNF367/cell cycle signaling pathway. Neuropsychiatr Dis Treat.

[48] Wang B, Zhao C-H, Sun G, Zhang Z-W, Qian B-M, Zhu Y-F (2019). IL-17 induces the proliferation and migration of glioma cells through the activation of PI3K/Akt1/NF-κB-p65. Cancer Lett.

[49] Wei L, Li L, Liu L, Yu R, Li X, Luo Z (2021). Knockdown of annexin-A1 inhibits growth, migration and invasion of glioma cells by suppressing the PI3K/Akt signaling pathway. ASN Neuro.

[50] Su X, Yang Y, Guo C, Zhang R, Sun S, Wang Y (2021). NOX4-derived ROS mediates TGF-β1-induced metabolic reprogramming during epithelial-mesenchymal transition through the PI3K/AKT/HIF-1α pathway in glioblastoma. Oxid Med Cell Longev.

[51] Sun X, Chen Y, Tao X, Zhang W, Wang X, Wang X (2022). INPP4B inhibits glioma cell proliferation and immune escape via inhibition of the PI3K/AKT signaling pathway. Front Oncol.

[52] Pickup MW, Mouw JK, Weaver VM (2014). The extracellular matrix modulates the hallmarks of cancer. EMBO Rep.

[53] Gao S, Yang D, Fang Y, Lin X, Jin X, Wang Q (2019). Engineering Nanoparticles for Targeted Remodeling of the Tumor Microenvironment to Improve Cancer Immunotherapy. Theranostics.

[54] Iozzo RV, Sanderson RD (2011). Proteoglycans in cancer biology, tumour microenvironment and angiogenesis. J Cell Mol Med.

[55] Ringer P, Colo G, Fässler R, Grashoff C (2017). Sensing the mechano-chemical properties of the extracellular matrix. Matrix Biol.

[56] Paluch EK, Aspalter IM, Sixt M (2016). Focal adhesion-independent cell migration. Annu Rev Cell Dev Biol.

[57] Niklasson M, Bergstrom T, Jarvius M, Sundstrom A, Nyberg F, Haglund C (2019). Mesenchymal transition and increased therapy resistance of glioblastoma cells is related to astrocyte reactivity. J Pathol.

[58] Kent LN, Leone G (2019). The broken cycle: E2F dysfunction in cancer. Nat Rev Cancer.

[59] Aravindan S, Natarajan M, Herman TS, Aravindan N (2013). Radiation-induced TNFalpha cross signaling-dependent nuclear import of NFkappaB favors metastasis in neuroblastoma. Clin Exp Metastasis.

[60] Sun W, Zou Y, Cai Z, Huang J, Hong X, Liang Q (2022). Overexpression of NNMT in glioma aggravates tumor cell progression: an emerging therapeutic target. Cancers.

[61] Golebiewska A, Bougnaud S, Stieber D, Brons NH, Vallar L, Hertel F (2013). Side population in human glioblastoma is non-tumorigenic and characterizes brain endothelial cells. Brain.

[62] Hambardzumyan D, Gutmann DH, Kettenmann H (2016). The role of microglia and macrophages in glioma maintenance and progression. Nat Neurosci.

[63] Chen X, Zhang L, Zhang IY, Liang J, Wang H, Ouyang M (2014). RAGE expression in tumor-associated macrophages promotes angiogenesis in glioma. Can Res.

[64] Silver DJ, Sinyuk M, Vogelbaum MA, Ahluwalia MS, Lathia JD (2016). The intersection of cancer, cancer stem cells, and the immune system: therapeutic opportunities. Neuro Oncol.

[65] Zhang C, Cheng W, Ren X, Wang Z, Liu X, Li G (2017). tumor purity as an underlying key factor in glioma. Clin Cancer Res.

[66] Ogbomo H, Cinatl J, Mody CH, Forsyth PA (2011). Immunotherapy in gliomas: limitations and potential of natural killer (NK) cell therapy. Trends Mol Med.

[67] Gu-Trantien C, Migliori E, Buisseret L, de Wind A, Brohee S, Garaud S (2017). CXCL13-producing TFH cells link immune suppression and adaptive memory in human breast cancer. JCI Insight..

[68] Tang Y, Zhang AXJ, Chen G, Wu Y, Gu W (2021). Prognostic and therapeutic TILs of cervical cancer—current advances and future perspectives. Mol Ther Oncolytics.

[69] Chen Z, Hambardzumyan D (2018). Immune microenvironment in glioblastoma subtypes. Front Immunol.

[70] Komohara Y, Horlad H, Ohnishi K, Fujiwara Y, Bai B, Nakagawa T (2012). Importance of direct macrophage-tumor cell interaction on progression of human glioma. Cancer Sci.

[71] Facciabene A, Peng X, Hagemann IS, Balint K, Barchetti A, Wang LP (2011). Tumour hypoxia promotes tolerance and angiogenesis via CCL28 and T(reg) cells. Nature.

[72] Wang Y-A, Li X-L, Mo Y-Z, Fan C-M, Tang L, Xiong F (2018). Effects of tumor metabolic microenvironment on regulatory T cells. Mol Cancer.

[73] Ooi YC, Tran P, Ung N, Thill K, Trang A, Fong BM (2014). The role of regulatory T-cells in glioma immunology. Clin Neurol Neurosurg.

[74] Su S, Chen J, Yao H, Liu J, Yu S, Lao L (2018). CD10+GPR77+ cancer-associated fibroblasts promote cancer formation and chemoresistance by sustaining cancer stemness. Cell.

[75] Erin N, Grahovac J, Brozovic A, Efferth T (2020). Tumor microenvironment and epithelial mesenchymal transition as targets to overcome tumor multidrug resistance. Drug Resist Updat.

[76] Liu T, Han C, Wang S, Fang P, Ma Z, Xu L (2019). Cancer-associated fibroblasts: an emerging target of anti-cancer immunotherapy. J Hematol Oncol.

[77] Liang J, Piao Y, Holmes L, Fuller GN, Henry V, Tiao N (2014). Neutrophils promote the malignant glioma phenotype through S100A4. Clin Cancer Res.

[78] Massara M, Persico P, Bonavita O, Poeta Mollica V, Locati M, Simonelli M (2017). Neutrophils in Gliomas. Front Immunol.

[79] Roberts EW, Broz ML, Binnewies M, Headley MB, Nelson AE, Wolf DM (2016). Critical role for CD103(+)/CD141(+) dendritic cells bearing CCR7 for tumor antigen trafficking and priming of T cell immunity in melanoma. Cancer Cell.

[80] Farhood B, Najafi M, Mortezaee K (2019). CD8(+) cytotoxic T lymphocytes in cancer immunotherapy: a review. J Cell Physiol.

[81] Reardon DA, Gokhale PC, Klein SR, Ligon KL, Rodig SJ, Ramkissoon SH (2016). Glioblastoma eradication following immune checkpoint blockade in an orthotopic. Immunocompet Model Cancer Immunol Res.

[82] Kim JE, Patel MA, Mangraviti A, Kim ES, Theodros D, Velarde E (2017). Combination therapy with anti-PD-1, Anti-TIM-3, and focal radiation results in regression of murine gliomas. Clin Cancer Res.

[83] Caccese M, Indraccolo S, Zagonel V, Lombardi G (2019). PD-1/PD-L1 immune-checkpoint inhibitors in glioblastoma: a concise review. Crit Rev Oncol Hematol.

[84] Chen M, Wang H, Guo H, Zhang Y, Chen L (2021). Systematic investigation of biocompatible cationic polymeric nucleic acid carriers for immunotherapy of hepatocellular carcinoma. Cancers.

[85] Syn NL, Teng MWL, Mok TSK, Soo RA (2017). De-novo and acquired resistance to immune checkpoint targeting. Lancet Oncol.

[86] Wing K, Onishi Y, Prieto-Martin P, Yamaguchi T, Miyara M, Fehervari Z (2008). CTLA-4 control over Foxp3+ regulatory T cell function. Science.

[87] Daud AI, Loo K, Pauli ML, Sanchez-Rodriguez R, Sandoval PM, Taravati K (2016). Tumor immune profiling predicts response to anti-PD-1 therapy in human melanoma. J Clin Invest.

[88] Powles T, Eder JP, Fine GD, Braiteh FS, Loriot Y, Cruz C (2014). MPDL3280A (anti-PD-L1) treatment leads to clinical activity in metastatic bladder cancer. Nature.

[89] El-Khoueiry AB, Sangro B, Yau T, Crocenzi TS, Kudo M, Hsu C (2017). Nivolumab in patients with advanced hepatocellular carcinoma (CheckMate 040): an open-label, non-comparative, phase 1/2 dose escalation and expansion trial. Lancet.

[90] Raeber ME, Rosalia RA, Schmid D, Karakus U, Boyman O (2020). Interleukin-2 signals converge in a lymphoid-dendritic cell pathway that promotes anticancer immunity. Sci Transl Med.

[91] Tumeh PC, Harview CL, Yearley JH, Shintaku IP, Taylor EJM, Robert L (2014). PD-1 blockade induces responses by inhibiting adaptive immune resistance. Nature.

[92] Langer CJ, Gadgeel SM, Borghaei H, Papadimitrakopoulou VA, Patnaik A, Powell SF (2016). Carboplatin and pemetrexed with or without pembrolizumab for advanced, non-squamous non-small-cell lung cancer: a randomised, phase 2 cohort of the open-label KEYNOTE-021 study. Lancet Oncol.

[93] Li H, Yan R, Chen W, Ding X, Liu J, Chen G (2021). Long non coding RNA SLC26A4-AS1 exerts antiangiogenic effects in human glioma by upregulating NPTX1 via NFKB1 transcriptional factor. FEBS J.

[94] Chen W, Li Q, Zhang G, Wang H, Zhu Z, Chen L (2020). LncRNA HOXA-AS3 promotes the malignancy of glioblastoma through regulating miR-455-5p/USP3 axis. J Cell Mol Med.

[95] Gao Y, Yu H, Liu Y, Liu X, Zheng J, Ma J (2018). Long non-coding RNA HOXA-AS2 regulates malignant glioma behaviors and vasculogenic mimicry formation via the MiR-373/EGFR axis. Cell Physiol Biochem.

[96] Shi T, Guo D, Xu H, Su G, Chen J, Zhao Z (2020). HOTAIRM1, an enhancer lncRNA, promotes glioma proliferation by regulating long-range chromatin interactions within HOXA cluster genes. Mol Biol Rep.

[97] Zhang S, Wan H, Zhang X (2020). LncRNA LHFPL3-AS1 contributes to tumorigenesis of melanoma stem cells via the miR-181a-5p/BCL2 pathway. Cell Death Dis.

[98] Donati B, Lorenzini E, Ciarrocchi A (2018). BRD4 and cancer: going beyond transcriptional regulation. Mol Cancer.

[99] Nishida J, Momoi Y, Miyakuni K, Tamura Y, Takahashi K, Koinuma D (2020). Epigenetic remodelling shapes inflammatory renal cancer and neutrophil-dependent metastasis. Nat Cell Biol.

[100] Kuras L, Borggrefe T, Kornberg RD (2003). Association of the Mediator complex with enhancers of active genes. Proc Natl Acad Sci USA.

[101] Loven J, Hoke HA, Lin CY, Lau A, Orlando DA, Vakoc CR (2013). Selective inhibition of tumor oncogenes by disruption of super-enhancers. Cell.

[102] Boija A, Klein IA, Sabari BR, Dall’Agnese A, Coffey EL, Zamudio AV (2018). Transcription factors activate genes through the phase-separation capacity of their activation domains. Cell.

