# Supplementary figures and images for: Integrative analysis of a novel super-enhancer-associated lncRNA prognostic signature and identifying LINC00945 in aggravating glioma progression
Source: Hum Genomics. 2023 Mar 31;17:33. doi: 10.1186/s40246-023-00480-w (PMC10064652; doi:10.1186/s40246-023-00480-w)

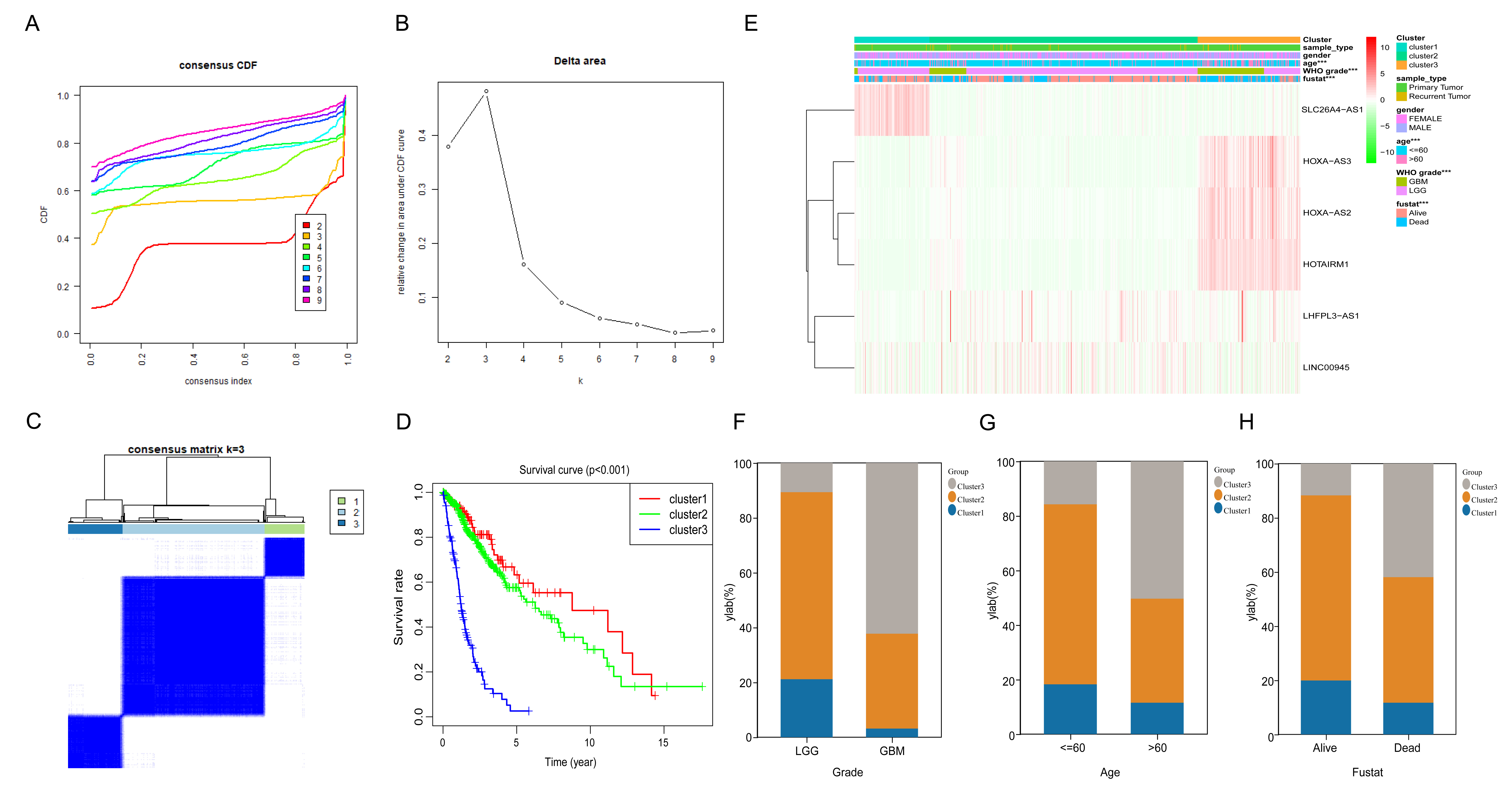

Supplement: Supplementary file 1 — Additional file 1: Fig. S1 6 SE-lncRNAs classified glioma patients into 3 clusters in the TCGA dataset. A CDF of consensus clustering for k = 2 to 9. B A relative change in area under the CDF curve was shown. C The consensus clustering matrix revealed that patients were divided into 3 clusters (k = 3). D Survival difference of patients in cluster 1/2/3 subgroups. E Heatmap of clinicopathologic distribution and 6 SE-lncRNAs expression levels among cluster 1/2/3. F–H The clinicopathological features of grade (F), age (G), and survival status (H) in 3 subgroups. ***p < 0.001. [file 40246_2023_480_MOESM1_ESM.jpg]

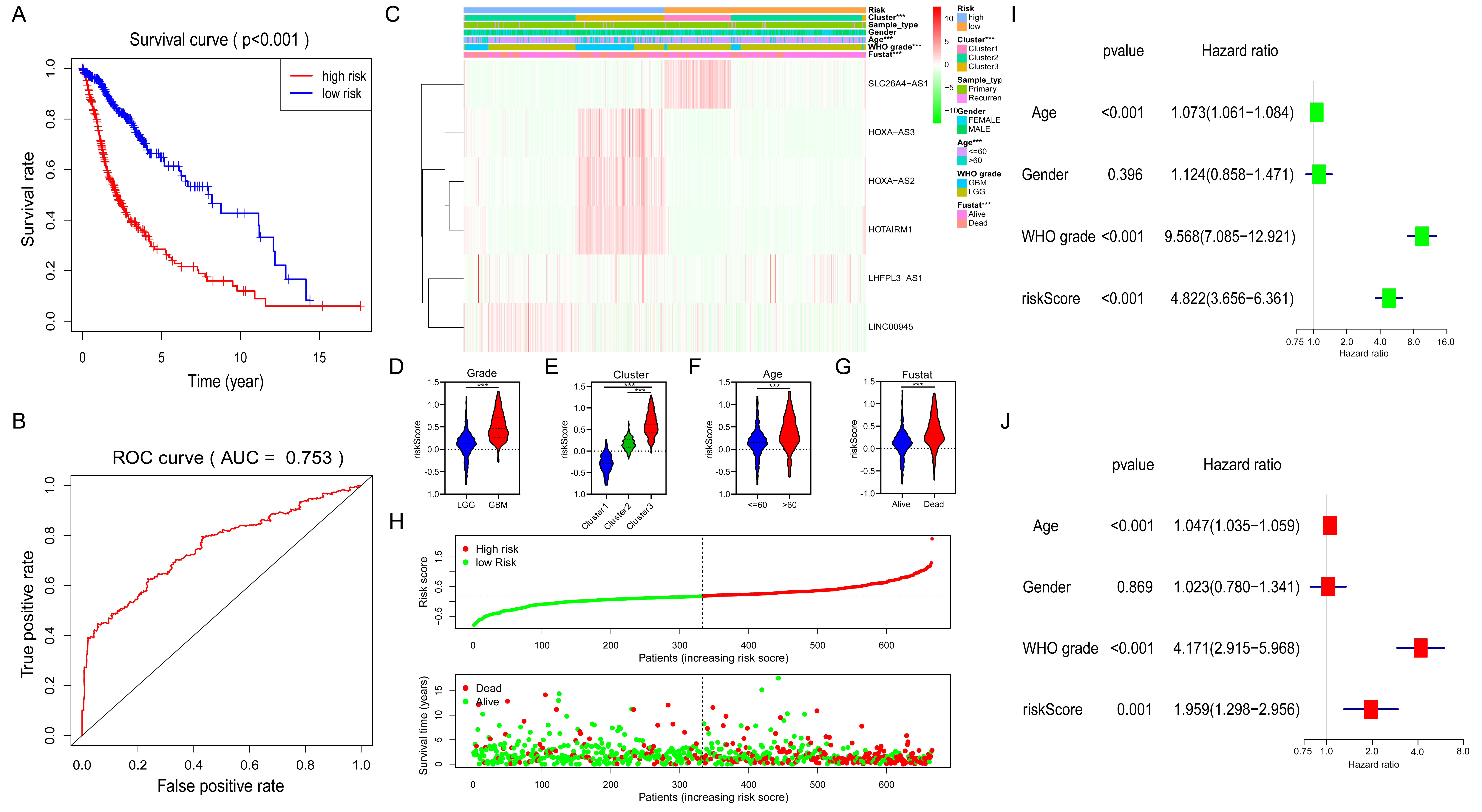

Supplement: Supplementary file 2 — Additional file 2: Fig. S2 Verification of risk model in TCGA dataset. A Kaplan-Meier OS curves for patients in two risk groups. B ROC curves presented the predictive efficiency of our risk model in the validation set. C The heatmap displayed the differential clinicopathological features and 6 SE-lncRNAs expression levels in two risk groups. D–G The WHO grade (D), cluster 1/2/3 subgroups (E), age (F), and fustat (G) stratified the validation set, and the distribution of risk scores was shown. H Distribution and survival differences of patients in two risk groups in the TCGA dataset. I, J Univariate (I) and multivariate (J) Cox analyses included age, gender, WHO grade, and risk score in the validation set. ***p < 0.001. [file 40246_2023_480_MOESM2_ESM.jpg]

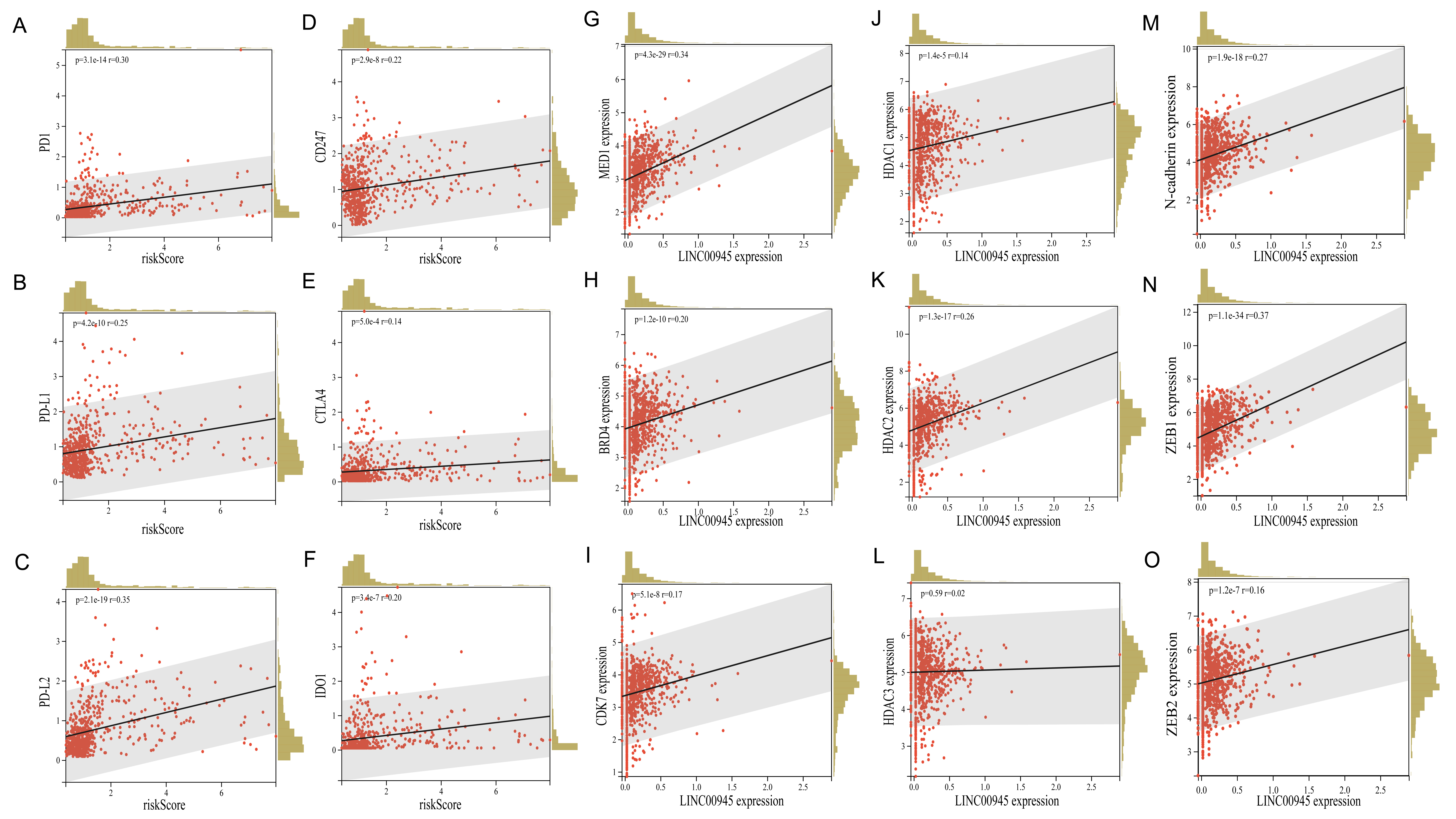

Supplement: Supplementary file 3 — Additional file 3: Fig. S3 The correlation analysis of risk score and immune checkpoint genes, and LINC00945 and super-enhancer related factors or EMT-related genes expression in CGGA dataset. A–F Correlation between risk scores and immune checkpoint genes, including PD1 (A), PD-L1 (B), PD-L2 (C), CD247 (D), CTLA4 (E), and IDO1 (F). G–L The correlation of LINC00945 with MED1 (G), BRD4 (H), CDK7 (I), HDAC1 (J), HDAC2 (K), and HDAC3 (L), respectively. M–O LINC00945 was positively related to the expression of N-cadherin (M), ZEB1 (N), and ZEB2 (O) in the CGGA database. [file 40246_2023_480_MOESM3_ESM.jpg]

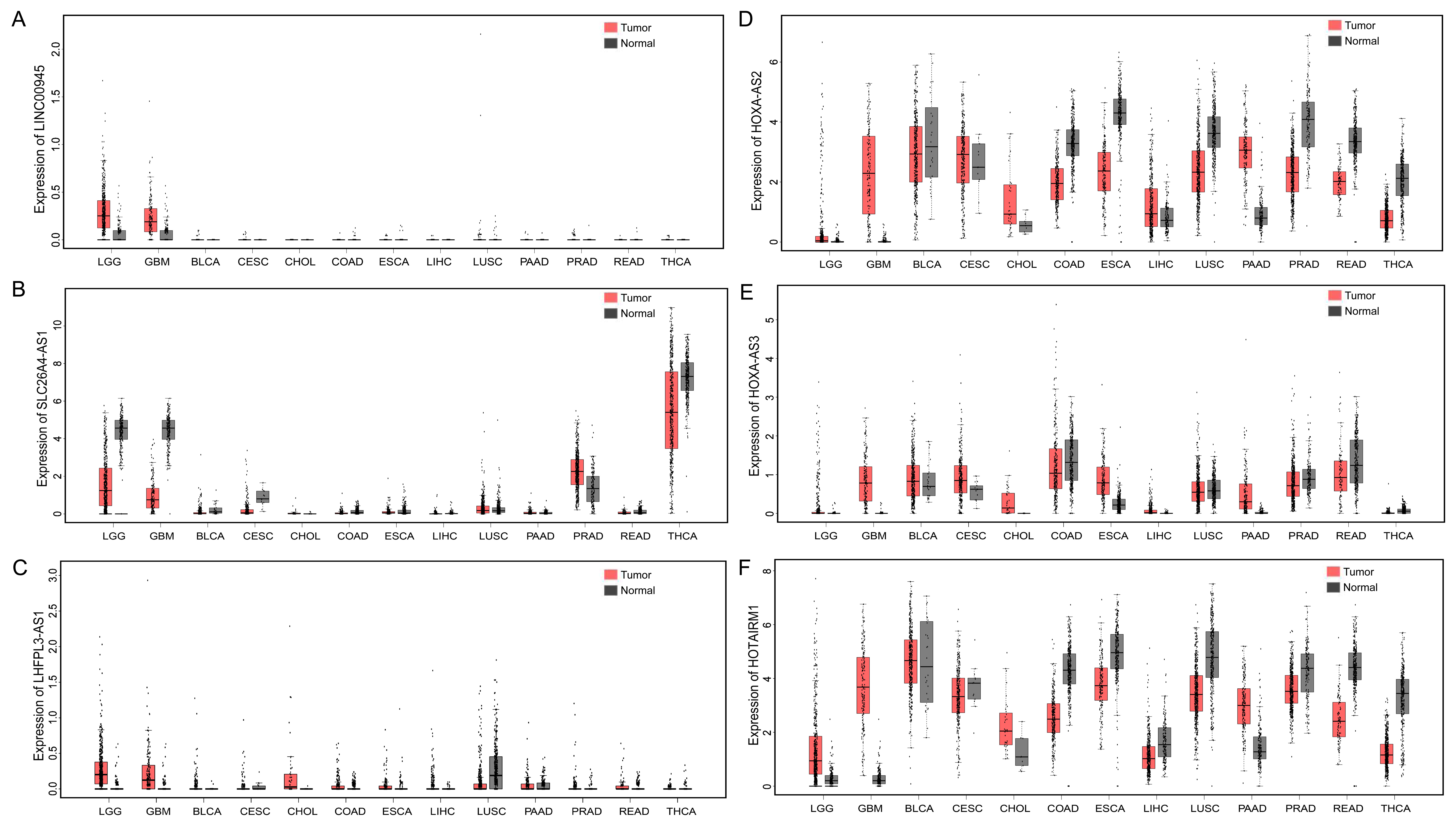

Supplement: Supplementary file 4 — Additional file 4: Fig. S4 The expression of LINC00945 (A), SLC26A4-AS1 (B), LHFPL3-AS1 (C), HOXA-AS2 (D), HOXA-AS3 (E), and HOTAIRM1 (F) in certain tumor type and normal tissue. [file 40246_2023_480_MOESM4_ESM.jpg]

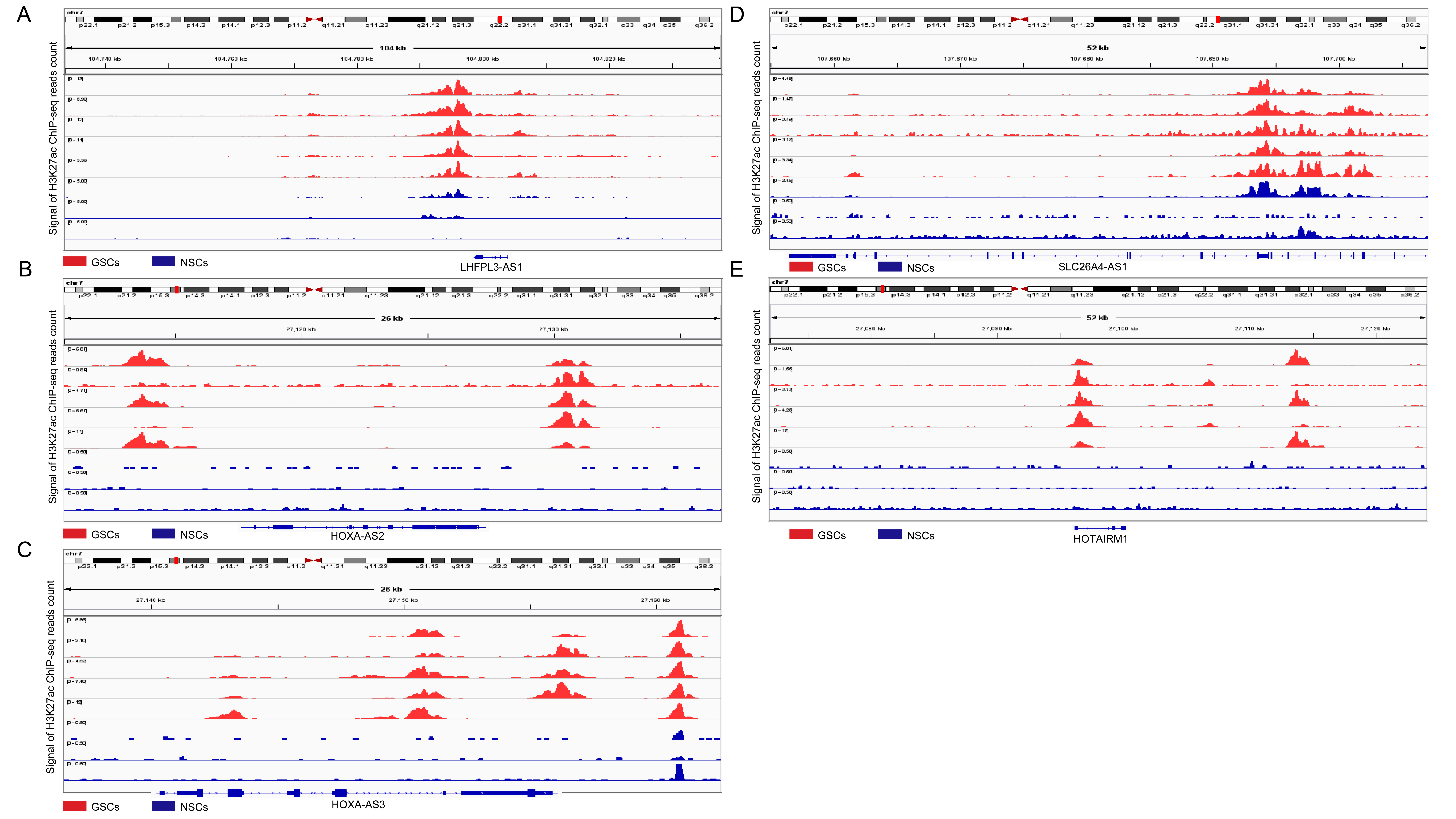

Supplement: Supplementary file 5 — Additional file 5: Fig. S5 H3K27ac ChIP-seq data identified SEs of LHFPL3-AS1 (A), HOXA-AS2 (B), HOXA-AS3 (C), SLC26A4-AS1 (D), and HOTAIRM1 (E) in GSCs. [file 40246_2023_480_MOESM5_ESM.jpg]
