# Supplementary material for: Integrative analysis of a novel super-enhancer-associated lncRNA prognostic signature and identifying LINC00945 in aggravating glioma progression
Source: Hum Genomics. 2023 Mar 31;17:33. doi: 10.1186/s40246-023-00480-w (PMC10064652; doi:10.1186/s40246-023-00480-w)
Supplement: Supplementary file 6 — Additional file 6: Table S1 Clinic-pathological characteristics of 12 glioma patients. Table S2 Primer pairs used for enhancer regions in ChIP-qPCR and performing RT-qPCR. [file 40246_2023_480_MOESM6_ESM.docx]

**Supplementary Table 1 |** Clinic-pathological characteristics of 12 glioma patients.

| Characteristic | number of patients |
| --- | --- |
|  |  |
| KPS |  |
| >80 | 9 |
| <80 | 3 |
| Gender |  |
| Male | 6 |
| Female | 6 |
| Age |  |
| ≤ 50 | 4 |
| > 50 | 8 |
| Grade |  |
| Low grade | 5 |
| GBM | 7 |

**Supplementary Table 2 |** Primer pairs used for enhancer regions in ChIP-qPCR and performing RT-qPCR.

|  | Forward Primer | Reverse Primer |
| --- | --- | --- |
| LINC00945-SE1 | AGACTCGCGTATCCAGTGTT | GGGGAAAGGGAAACGAAAGG |
| LINC00945-SE2 | CCACAGCCTTCCAAGCAAAT | GAAGAGTTGGTTTGGGGCAG |
| LINC00945-SE3 | AAGTCCCCTTCTGTGTCTCG | ATATGGGTCACTGAAGCGCT |
| MED1 | GAGAATCCTGTGAGCTGTCCG | GTTGCTTTCCAGTACATAATTGC |
| BRD4 | GTGGTGCACATCATCCAGTC | GCTTCAGGGTCTCAAAGTCG |
| LINC00945 | CAGAGCCACCTTTGTTTCCC | GAAAAGATGCGAGCGGTTCT |
| GAPDH | CGCTCTCTGCTCCTCCTGT | ATCCGTTGACTCCGACCTA |
